# Size-dependent tissue-specific biological effects of core–shell structured Fe_3_O_4_@SiO_2_–NH_2_ nanoparticles

**DOI:** 10.1186/s12951-019-0561-4

**Published:** 2019-12-23

**Authors:** Jinquan Li, Zhongxue Yuan, Huili Liu, Jianghua Feng, Zhong Chen

**Affiliations:** 1 School of Pharmaceutical Science (Shenzhen), Sun Yat-Sen University, Guangzhou, 510275 China; 2 Department of Electronic Science, Fujian Provincial Key Laboratory of Plasma and Magnetic Resonance, Xiamen University, 422 Siming South Road, Siming District, Xiamen, 361005 China; 3 State Key Laboratory of Magnetic Resonance and Atomic and Molecular Physics, Wuhan Center for Magnetic Resonance, Wuhan Institute of Physics and Mathematics, Chinese Academy of Sciences, Wuhan, 430071 China

**Keywords:** Core–shell structure nanoparticle, Size effect, Untargeted metabolomics, Toxicity assessment

## Abstract

**Background:**

Understanding the in vivo size-dependent pharmacokinetics and toxicity of nanoparticles is crucial to determine their successful development. Systematic studies on the size-dependent biological effects of nanoparticles not only help to unravel unknown toxicological mechanism but also contribute to the possible biological applications of nanomaterial.

**Methods:**

In this study, the biodistribution and the size-dependent biological effects of Fe_3_O_4_@SiO_2_–NH_2_ nanoparticles (Fe@Si-NPs) in three diameters (10, 20 and 40 nm) were investigated by ICP-AES, serum biochemistry analysis and NMR-based metabolomic analysis after intravenous administration in a rat model.

**Results:**

Our findings indicated that biodistribution and biological activities of Fe@Si-NPs demonstrated the obvious size-dependent and tissue-specific effects. Spleen and liver are the target tissues of Fe@Si-NPs, and 20 nm of Fe@Si-NPs showed a possible longer blood circulation time. Quantitative biochemical analysis showed that the alterations of lactate dehydrogenase (LDH) and uric acid (UA) were correlated to some extent with the sizes of Fe@Si-NPs. The untargeted metabolomic analyses of tissue metabolomes (kidney, liver, lung, and spleen) indicated that different sizes of Fe@Si-NPs were involved in the different biochemical mechanisms. LDH, formate, uric acid, and GSH related metabolites were suggested as sensitive indicators for the size-dependent toxic effects of Fe@Si-NPs. The findings from serum biochemical analysis and metabolomic analysis corroborate each other. Thus we proposed a toxicity hypothesis that size-dependent NAD depletion may occur in vivo in response to nanoparticle exposure. To our knowledge, this is the first report that links size-dependent biological effects of nanoparticles with in vivo NAD depletion in rats.

**Conclusion:**

The integrated metabolomic approach is an effective tool to understand physiological responses to the size-specific properties of nanoparticles. Our results can provide a direction for the future biological applications of Fe@Si-NPs.

## Background

Iron oxide nanoparticles (IONPs) are magnetic, relatively non-toxic and highly biocompatible, thus giving IONPs potential biomedical applications in a wide range of fields such as diagnostic imaging [1–3], drug delivery [4], gene therapy [5], magnetic nanoparticle separation [6], in vivo cell tracking [7], or as iron supplement for the treatment of metal poisoning [8]. However, due to the high surface energies and the magnetic dipole–dipole attractions between the nanoparticles, IONPs are highly prone to agglomeration under physiological conditions and nonspecific adsorption of biomolecules [9]. Coating IONPs with silica is considered as an effective means to solve their dispersibility and adsorption [10]. Silica-coated core–shell nanoparticles showed sevenfold clearer contrasts in magnetic resonance imaging than simple nanoparticles [11]. However, it has been proved that silanol groups on the plain silica coated IONPs play a major role in nanoparticle-cell interactions and cause membrane protein degradation and intracellular free radical production [12, 13]. Thus numerous surface functionalization methods have been introduced to improve the biocompatibility of silica-coated IONPs.

Recently, amino-functionalized Fe_3_O_4_@SiO_2_ nanoparticles (Fe@Si-NPs), taking advantage of their functional synergistic effects from the different components, demonstrated outstanding biocompatibility and special magnetic properties compared with the existing Fe_3_O_4_@SiO_2_ nanoparticles [14, 15]. The silica shells not only prevent the oxidation and agglomeration of magnetic core at physiological conditions but also enhance their chemical stability. The amino modifications of the silica surface decrease the detrimental interactions with cellular membranes and prolong the blood circulation time after in vivo administration. Studies have shown that the functionalized silanol moieties with amino groups on the surface of silica-coated IONPs can effectively reduce toxicity both in vitro and in vivo, and render the nanoparticle great biocompatibility [16]. Thus, they show promising biological applications such as biomedical imaging, gene delivery, and drug transport [12, 16].

Besides the surface modification and chemistry, the particle size is also a significant factor for tuning the biodistribution and the potential toxicity of nanoparticles, which can deeply affect their pharmacokinetics, namely absorption and internalization, biodistribution, metabolic fate and elimination from the body, as well as their performance. As a rule of thumb, nanoparticles between 10 and 100 nm in diameter can avoid prompt spleen, liver and kidney filtration, and extend the blood circulation time and increase the access of nanoparticles to the targeted organ. The biodistribution and accumulation of iron oxide nanoparticles in various tissues/organs can be indirectly evaluated by the determination of whole iron content using inductively coupled plasma spectroscopy or Prussian blue staining [17]. On the other hand, due to their intrinsic complexity, most of the toxicity results of core–shell IONPs are based on in vitro assays, e.g. Urbas et al. [18] demonstrated that IONP size around 100 nm can affect cell metabolism, while Majeed et al. [19] reported that IONPs with sizes around 10–20 nm do not have obvious toxic effect. Although these researches provided useful preliminary information and suggested that toxicities are in inverse proportion to the size of nanoparticles, these results may not be applicable for all the other cell types or organs in the body [17, 20]. Most importantly, there is lack of correlation between the in vivo biodistribution and the in vitro toxic effects, and there is no clear understanding of the size effect in vivo.

Drugs or toxins can cause the concentration variations of endogenous metabolites and thus shift the fluxes of metabolic pathways. Monitoring the metabolic changes in different organs will provide direct information on the functioning of biochemical pathways and the possible harmful effects of these nanoparticles. Therefore a detailed metabolic response of different size core–shell IONPs in organs is of prime importance for next phase of clinical studies. In drug discovery process, metabolomic analysis can be used to monitor drug side-effect and metabolism, identify biomarkers, explore the mechanism of action, and provide the feedback on the tissue-specificity [21]. It has been proved to be an effective method to evaluate the nanotoxicity in the previous study [22].

After completing the evaluation of the subchronic toxic effects of 20 nm Fe@Si-NPs, we obtained the information on dose selection, one critical factor in developing biomedical nanomaterials [23]. However, the size effects of Fe@Si-NPs, another critical factor associated with toxicity, still need to be assessed. The in vivo performance of IONPs usually achieved optimization in dozens of minutes or hours following intravenous injection, and the potential adverse effects of contrast agent administration, such as post-contrast acute kidney injury (PC-AKI), mainly occur within 48 h after injection [24]. This means that the first 48 h is critical for toxicological assessment. Therefore, we chose 48 h and 6 h (an early stage of the acute-phase response) to investigate the acute toxic effects of Fe@Si-NPs.

On toxicokinetics, previous report showed that the small IONPs (around 10 nm) were more easily taken up by the liver and rapidly cleared by kidneys, while the large IONPs (around 40 nm) mainly by the spleen [25]. The diameters of the related commercial products are usually of about 20 nm, such as Feridex (< 30 nm) and Resovist (around 17 nm). In this study, in view of the different structure between IONPs and Fe@Si-NPs, we chose the bilateral sizes (10 nm and 40 nm) of the typical diameter of IONPs (20 nm) for optimization. A combined analysis of serum biochemical parameters, metabolomic data and the iron content of the target tissues was applied to uncover the correlation between biodistribution and acute toxic effects of Fe@Si-NPs, provide comprehensive and complementary insights into their size effects in vivo, and lead to toxicity hypothesis generation.

## Materials and methods

### Synthesis and characterization of core–shell structured nanoparticles

Fe@Si-NPs were prepared by following the multistep synthetic procedure as reported previously [14]. Briefly, in the first step, monodisperse magnetic Fe_3_O_4_ cores (around 2.3 to 4.0 nm in diameter) were synthesized by a modified solvothermal method. In the second step, according to the Stober method, three different thicknesses of silica shell were coated on the magnetic core by adjusting the concentration ratio of ammonium to tetraethyl orthosilicate [26]. In the third step, the obtained core–shell structures of three different sizes (around 10, 20 nm, and 40 nm in diameter) were respectively functionalized with *N*-(2-aminoethyl)-3-aminopropyl trimethoxysilane (AEAPS) to introduce amino groups by sol–gel co-condensation method. Afterward the precipitate was collected, washed, dried in vacuum, and characterize by TEM (Additional file 1: Figure S1). Before use, Fe@Si-NPs were freshly well-dispersed by ultrasound in saline solution.

### Animal handling and biological sample collection

All animals involved in this study were cared for according to the principles of the National Institutes of Health Guide for the Care and Use of Laboratory Animals and approved by the Ethical and Research Committee of Xiamen University (SYXK 2013-0006). All animal experiments were performed at specific pathogen free (SPF) facility of Xiamen University Laboratory Animal Center (XMULAC). A total of 52 9-week-old male Sprague Dawley rats (258 ± 10 g) were used in our study. The environment conditions were set at 21–26 °C with a relative humidity of 45–70%, and a 12/12-h light/dark cycle. Food and tap water were provided ad libitum. After 2 week of acclimatization, these 52 rats were randomly assigned to four groups (control group, 10 nm, 20 nm, and 40 nm Fe@Si-NPs-exposure groups, 13 rats each group), and a single dose of Fe@Si-NPs in saline was administrated intravenously to the rats at dose of 1 mg Fe/kg body weight (b. w.). Control group was treated with saline only. Animals were sacrificed by exsanguination under isoflurane anesthesia at time point on 6 h (5 rats each group) post-dose (p. d.) and 48 h (8 rats each group) p. d. Blood sample (1 mL) was collected for serum biochemical profile analysis. Kidney, liver, lung, and spleen tissues were excised in duplicate and snap-frozen in liquid nitrogen for tissue extraction and ICP-AES analysis. These samples were stored at − 80 °C until use.

### Serum biochemical analysis

Standard spectrophotometric methods were carried out to measure the following biochemical parameters on a Roche Modular P800 automatic analyzer (Roche Diagnostics, Germany): total protein (TP), albumin (Alb), globulin (Glo), Alb/Glo, total bilirubin (Tbil), direct bilirubin (Dbil), indirect bilirubin (Ibil), alanine aminotransferase (ALT), aspartate aminotransferase (AST), AST/ALT, gamma glutamyltransferase (GGT), alkaline phosphatase (ALP), triglycerides (TG), total cholesterol (TC), high density lipoprotein (HDL), low density lipoprotein (LDL), glucose (Glu), lactate dehydrogenase (LDH), blood urea nitrogen (Bun), creatinine (Cn), Bun/Cn, uric acid (UA), and total bile acid (TBA). All parameters are expressed as mean ± standard deviation (SD).

### Sample preparation and ^1^H NMR spectroscopic analysis

The method for the extraction of polar metabolites was established by previous researchers [27]. In brief, pre-weighed kidney, liver, lung, or spleen sample (100 mg) was homogenized in 400 μL of CH_3_OH and 85 μL of H_2_O at 4 °C. The homogenates were transferred into a 2.5-mL tube, and mixed with 400 μL of CHCl_3_ and 200 μL of H_2_O and vortexed for 60 s. After 10 min partitioning on ice, the samples were centrifuged for 5 min (10,000×*g*, 4 °C). The upper supernatants were transferred into 1.5 mL tubes, and lyophilized to remove CH_3_OH and H_2_O. The extracts were reconstituted in 0.5 mL D_2_O containing 1 mM TSP, then transferred into 5 mm NMR tubes and analyzed by NMR spectroscopy. ^1^H NMR spectra of these samples were acquired on a Bruker-AV600 spectrometer at 296 K. Standard 1D ^1^H spectra were acquired with a NOESPYPR1D pulse sequence. For each sample, 64 FIDs were collected into 32 K data points over a spectral width of 12 kHz with a relaxation delay of 6.5 μs and an acquisition time of 2.66 s.

### Spectral processing and data multivariate analyses

The FIDs were multiplied by an exponential function corresponding to a 1 Hz line-broadening factor before Fourier transform to increase the signal-to-noise ratio. The acquired NMR spectra were manually phase- and baseline-corrected using MestReNova (V9.0, Mestrelab Research, Santiago de Compostela, Galicia, Spain) and referenced to TSP at δ 0.00 for tissue extract samples. The segments of δ 5.12–4.57 and δ 3.37–3.35 in the spectra were excluded to remove variation in residual water and methanol signal. The remainder spectral region δ 9.5–0.5 was segmented into regions of 0.001 ppm. Normalization was applied to the data from each sample, which made the data directly comparable with each other.

SIMCA v14.0 (Umetrics, Umea, Sweden) was served to multivariate statistical analysis. Unsupervised principal component analysis (PCA) was performed using a mean-centered scaling to verify the quality of spectra and visualize the grouping of the samples. Supervised orthogonal partial least-squares discriminant analysis (OPLS-DA) was carried out at a Pareto scaling to identify differences between groups.

The results were visualized in the forms of scores plots to show the separation between groups and volcano plots to visualize − log_2_ of fold changes in concentration on the x-axis versus the − log_10_ of the P-value on the y-axis. In the volcano plots, the color was associated with the correlation coefficients, hot color for strong correlation, and cool color for weak correlation, and the size of dot was associated to VIP value. Both the correlation coefficients and VIP values were derived from OPLS-DA. Positive log_2_ values of fold changes stand for the metabolites in up in comparison to the control group.

The cutoff levels for correlation coefficients, VIP values, and *P*-values for the univariate statistical analysis were | r | > 0.666 (the degree of freedom equals to 4), the top 20% of all VIP scores, and P value < 0.05, respectively. The metabolites, met at least two of three criteria, were selected as the discriminatory ones.

### Statistical analysis

Statistical analyses were performed using SPSS software (version 21.0 for Windows; SPSS, Inc., Chicago, IL, USA). Significant differences between the experimental groups and corresponding control group were analyzed by using the unpaired two-tailed Student’s *t*-test. The *P* values less than 0.05 were considered statistically significant. The results were expressed as mean ± SD.

## Results

### Effects of nanoparticle size on iron biodistribution in tissues of Fe@Si-NPs

**Fig. 1:**
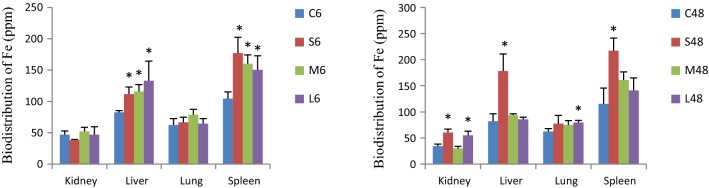
Biodistribution of Fe in the different tissues in the different dose groups at 6 h and 48 h post-administration of Fe@Si-NPs. C, control group; S, small-size group; M, mid-size group; L, large-size group; 6, 6 h post-dose; 48, 48 h post-dose. *Significant differences between treatment and control groups as judged by Student’s t-test (*P < 0.05)

We employed ICP-AES to determine the iron concentration in tissues as an indirect assessment for the biodistribution of Fe@Si-NPs. On the whole, as shown in Fig. 1, the highest uptakes of all the three sizes of nanoparticles were concentrated in spleen and liver at 6 h p.d., whereas the iron contents of spleen and liver in the mid- and large-size groups displayed a significant decrease at 48 h p.d. The kidney uptake of small-size and large-size Fe@Si-NPs took place at 48 h p.d., while the mid-size Fe@Si-NPs showed no tissue specific infiltration at 48 h p.d. suggesting that they would have longer blood circulation time.

The iron contents of liver in the small-, mid- and large-size groups were increased to 135% (P < 0.05), 140% (P < 0.05), and 161% (P < 0.05), and those of spleen were increased to 169% (P < 0.05), 153% (P < 0.05) and 143% (P < 0.05) of the corresponding controls, respectively. However, no significant distribution difference of iron in kidneys and lung was observed at 6 h p. d. At 48 h p. d., the iron content of kidney in the small- and large-size groups was increased to 176% (P < 0.05) and 159% (P < 0.05) of the controls, respectively, and the iron content in liver (small-size group), lung (large-size group) and spleen (small-size group) was increased to 217% (P < 0.05), 128% (P < 0.05) and 188% (P < 0.05) of the controls, respectively, but no significant distribution difference of iron contents in other groups was observed. These results suggest that all three sizes of Fe@Si-NPs tend to accumulate in the liver and spleen at 6 h p. d., however, 48 h after treatment, the small-size Fe@Si-NPs are sequestered not only by liver and spleen but also by kidneys; and the large-size ones reach kidney and lung.

In addition, there was no statistically significant difference in the ratio of the tissue weight to the total body weight of rats between all Fe@Si-NPs-treated groups and the corresponding controls (Additional file 1: Figure S2). Thus, the iron concentration measured with ICP-AES positively correlative to the biodistribution and tissue retention of Fe@Si-NPs. In addition, no apparent pathological change was observed in the kidney, lung, liver and spleen (Additional file 1: Figure S3).

### Effects of nanoparticle size of Fe@Si-NPs on the serum biochemical parameters

There are a wide variety of substances in serum, including proteins, enzymes, lipids, metabolites etc. Testing for these substances provides information on the functional status of tissues in the body. In this study, serum biochemical analyses were performed to quantitatively evaluate the effects of three different size nanoparticles exposure on rats. Table 1 shows that the levels of Glo, Alb/Glo, Ibil, AST/ALT, TG, TC, LDL-C, Glc, LDH, BUN, Cn, BUN/Cn, and UA in rat sera in some treatment groups changed distinctly compared to the corresponding controls, however, only the changes of LDH and UA levels were, respectively negatively and positively, correlated with the sizes of Fe@Si-NPs to some extent. As shown in Table 1, a decrease of LDH levels to 87%, 63% (P < 0.001), and 48% (P < 0.001) of the controls at 6 h p. d., and 88%, 69%, and 66% of the controls at 48 h p. d. was observed in small-, mid- and large-size Fe@Si-NPs treated groups, respectively. On the contrary, an increase of UA levels to 140% (P < 0.001), 164% (P < 0.001), and 173% (P < 0.001) of the controls at 6 h p. d., and 116%, 138% (P < 0.001), and 127% (P < 0.05) of the controls at 48 h p. d. was observed in small-, mid- and large-size Fe@Si-NPs treated groups, respectively.

**Table 1:** Effect of Fe_3_O_4_@SiO_2_–NH_2_ nanoparticle administration on blood biochemical indexes

Indexes	C6^a^	S6	M6	L6	C48	S48	M48	L48
TP (g/L)	54.96 ± 2.18^b^	55.44 ± 2.29	57.56 ± 3.38	57.62 ± 1.60	55.53 ± 1.98	56.71 ± 2.36	55.04 ± 2.26	56.30 ± 3.59
Alb (g/L)	30.66 ± 1.01	30.54 ± 0.74	30.98 ± 1.08	30.74 ± 0.35	30.95 ± 0.75	30.86 ± 0.88	29.81 ± 0.75*	30.51 ± 1.25
Glo (g/L)	24.30 ± 1.24	24.90 ± 1.69	26.58 ± 2.37	26.88 ± 1.35*	24.58 ± 1.43	25.85 ± 1.62	25.23 ± 1.63	25.79 ± 2.36
Alb/Glo	1.26 ± 0.03	1.23 ± 0.06	1.17 ± 0.07*	1.15 ± 0.05**	1.26 ± 0.06	1.2 ± 0.05	1.19 ± 0.05*	1.19 ± 0.06
Tbil (μmol/L)	2.14 ± 0.23	2.36 ± 0.15	2.06 ± 0.25	2.24 ± 0.45	2.22 ± 0.19	2.40 ± 0.21	2.39 ± 0.16	2.39 ± 0.21
Dbil (μmol/L)	0.80 ± 0.07	0.72 ± 0.08	0.78 ± 0.11	0.78 ± 0.08	0.82 ± 0.08	0.86 ± 0.09	0.85 ± 0.09	0.88 ± 0.10
Ibil (μmol/L)	1.34 ± 0.18	1.64 ± 0.09*	1.28 ± 0.25	1.46 ± 0.40	1.40 ± 0.15	1.54 ± 0.14	1.54 ± 0.09	1.51 ± 0.12
ALT (U/L)	56.20 ± 5.81	51.20 ± 5.36	65.80 ± 8.41	76.80 ± 33.07	49.17 ± 8.86	46.88 ± 6.33	48.13 ± 9.72	45.88 ± 3.40
AST (U/L)	204.40 ± 36.05	195.20 ± 33.52	202.60 ± 18.94	195.60 ± 60.80	159.50 ± 45.05	139.25 ± 33.78	135.25 ± 26.76	133.75 ± 23.87
AST/ALT	3.64 ± 0.51	3.88 ± 0.93	3.10 ± 0.35	2.66 ± 0.40*	3.23 ± 0.59	2.96 ± 0.47	2.85 ± 0.45	2.91 ± 0.46
GGT (U/L)	0.64 ± 0.49	0.28 ± 0.40	0.28 ± 0.40	0.46 ± 0.49	1.00 ± 0.00	0.55 ± 0.48	0.89 ± 0.32	1.00 ± 0.00
ALP (U/L)	414.00 ± 119.44	338.00 ± 38.86	353.60 ± 20.79	345.20 ± 34.54	363.33 ± 77.29	320.5 ± 68.26	308.75 ± 61.68	291.38 ± 46.15
TG (mmol/L)	1.53 ± 0.35	1.49 ± 0.40	1.73 ± 0.65	2.23 ± 1.88	1.77 ± 0.62	1.76 ± 0.65	1.16 ± 0.33	0.91 ± 0.25**
TC (mmol/L)	2.49 ± 0.36	2.25 ± 0.42	2.54 ± 0.43	3.06 ± 0.35*	2.19 ± 0.18	2.42 ± 0.28	2.24 ± 0.25	2.48 ± 0.22**
HDL-C (mmol/L)	0.78 ± 0.10	0.68 ± 0.14	0.73 ± 0.09	0.80 ± 0.22	0.71 ± 0.07	0.75 ± 0.10	0.69 ± 0.11	0.77 ± 0.07
LDL-C (mmol/L)	0.27 ± 0.05	0.26 ± 0.07	0.34 ± 0.13	0.48 ± 0.10^**^	0.20 ± 0.03	0.25 ± 0.04*	0.26 ± 0.03*	0.25 ± 0.05
Glc (mmol/L)	5.19 ± 0.41	5.94 ± 0.26^**^	5.28 ± 0.77	5.93 ± 0.37*	5.64 ± 0.68	5.46 ± 0.50	6.03 ± 0.61	6.00 ± 0.37
LDH (U/L)	1436.50 ± 141.95	1243.36 ± 255.38	910.08 ± 165.81^**^	683.84 ± 202.51^**^	1143.87 ± 415.51	1007.39 ± 324.97	790.78 ± 253.54	749.79 ± 285.97
BUN (mmol/L)	4.88 ± 0.86	4.74 ± 0.93	4.00 ± 0.64	5.01 ± 0.60	4.76 ± 0.62	4.92 ± 0.39	5.70 ± 0.52*	4.96 ± 0.69
Cn (μmol/L)	46.60 ± 1.14	45.40 ± 4.83	44.60 ± 0.55^**^	45.20 ± 1.92	44.67 ± 1.97	44.00 ± 1.85	43.75 ± 2.25	45.75 ± 2.66
BUN/Cn	0.11 ± 0.02	0.11 ± 0.01	0.09 ± 0.02	0.11 ± 0.02	0.11 ± 0.02	0.11 ± 0.01	0.13 ± 0.01*	0.11 ± 0.02
UA (μmol/L)	89.00 ± 15.81	124.60 ± 8.68^**^	145.60 ± 14.93^**^	154.40 ± 17.14^**^	96.17 ± 17.01	111.75 ± 18.97	132.88 ± 14.26**	122.25 ± 25.79*
TBA (μmol/L)	21.78 ± 7.62	21.44 ± 5.74	18.82 ± 5.01	30.98 ± 7.49	21.97 ± 7.26	24.01 ± 6.79	28.9 ± 19.12	23.93 ± 9.87

*TP* total protein, *Alb* albumin, *Glo* globulin, *Tbil* total bilirubin, *Dbil* direct bilirubin, *Ibil* indirect bilirubin, *ALT* alanine aminotransferase, *AST* aspartate aminotransferase, *GGT* gamma glutamyltransferase, *ALP* alkaline phosphatase, *TG* triglycerides, *TC* total cholesterol, *HDL* high-density lipoprotein, *LDL* low-density lipoprotein, *Glc* glucose, *LDH* lactate dehydrogenase, *BUN* blood urea nitrogen, *Cn* creatinine, *UA* uric acid, *TBA* total bile acid

* Significant differences between treatment and control groups as judged by Student’s *t*-test using SPSS (*P < 0.05; **P < 0.001)

^a^C, control group; S, small-size group (around 10 nm in diameter); M, mid-size group (around 20 nm in diameter); L, large-size group (around 40 nm in diameter); 6, 6 h post-dose; 48,48 h post-dose

^b^Each value data represents the mean ± S.D

### H NMR spectral profiles and metabolic characteristics of tissues from rats

**Fig. 2:**
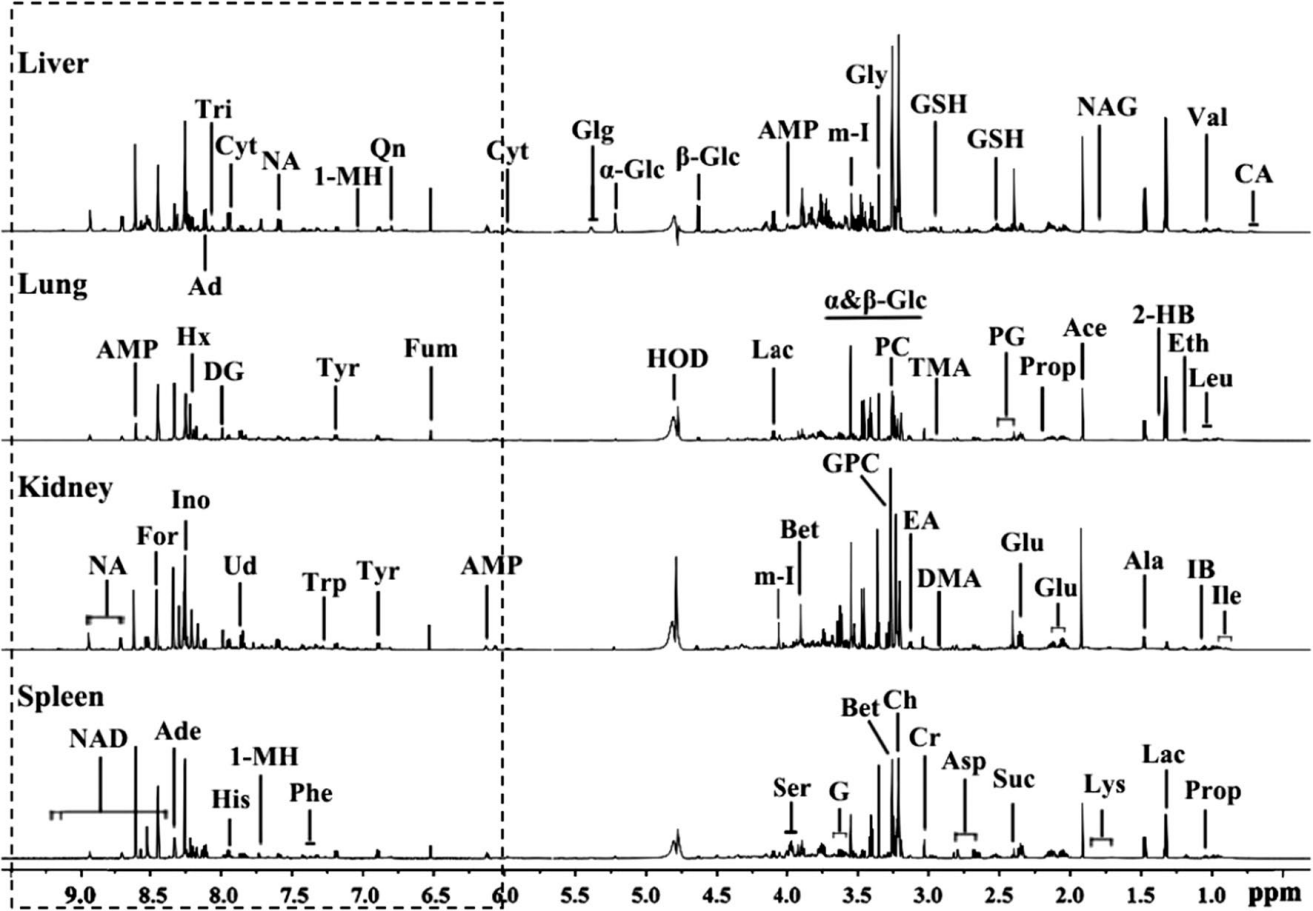
Typical ^1^H NMR spectra of deuterated liver, lung, kidney and spleen extracts from a low-dosed rat at 6 h p. d. The regions of δ 6.0–10.0 (in the dashed box) in the spectra were magnified 20 times in vertical expansion compared with the corresponding regions of δ 0.5–6.0. *Ace* acetate, *Ade* adenosine, *Ala* alanine, *AMP* adenosine monophosphate, *Asp* aspartate, *Bet* betaine, *Ch* choline, *Cr* creatine, *Cyt* cytidine, *DG* deoxyguanosine, *DMA* dimethylamine, *EA* ethanolamine, *Eth* ethanol, *For* formate, *Fum* fumarate, *G* glycerol, *Glg* glycogen, *α-Glc* alpha-glucose, *β-Glc* beta-glucose, *Glu* glutamate, *Gly* glycine, *GPC* glycerophosphocholine, *GSH* glutathione, *2-HB* 2-hydroxybutyrate, *His* histidine, *Hx* hypoxanthine, *m-I* myo-Inositol, *IB* isobutyrate, *Ile* isoleucine, *Ino* inosine, *Lac* lactate, *Leu* leucine, *Lys* lysine, *1-MH* 1-methylhistidine, *NA* nicotinamide, *NAD* NAD+, *NAG* *N*-acetylglutamate, *PC* phosphocholine, *PG* pyroglutamate, *Phe* phenylalanine, *Prop* propionate, *Qn* quinone, *Ser* serine, *Suc* succinate, *TMA* trimethylamine, *Tri* trigonelline, *Trp* tryptophan, *Tyr* tyrosine, *Ud* uridine, val valine

Representative ^1^H NMR spectra of tissue extracts including kidney, liver, lung and spleen are shown in Fig. 2. The primary peaks in the spectra were assigned to specific metabolites (Additional file 1: Table S1) according to previous studies [22, 23], and confirmed by a public NMR database (Human Metabolome Database V3.0, see www.hmdb.ca) and an in-house developed NMR database. Different tissues demonstrate their own unique spectral profiles, which provide the characteristic biochemical alterations corresponding to the specific tissues following the administration of Fe@Si-NPs.

**Fig. 3:**
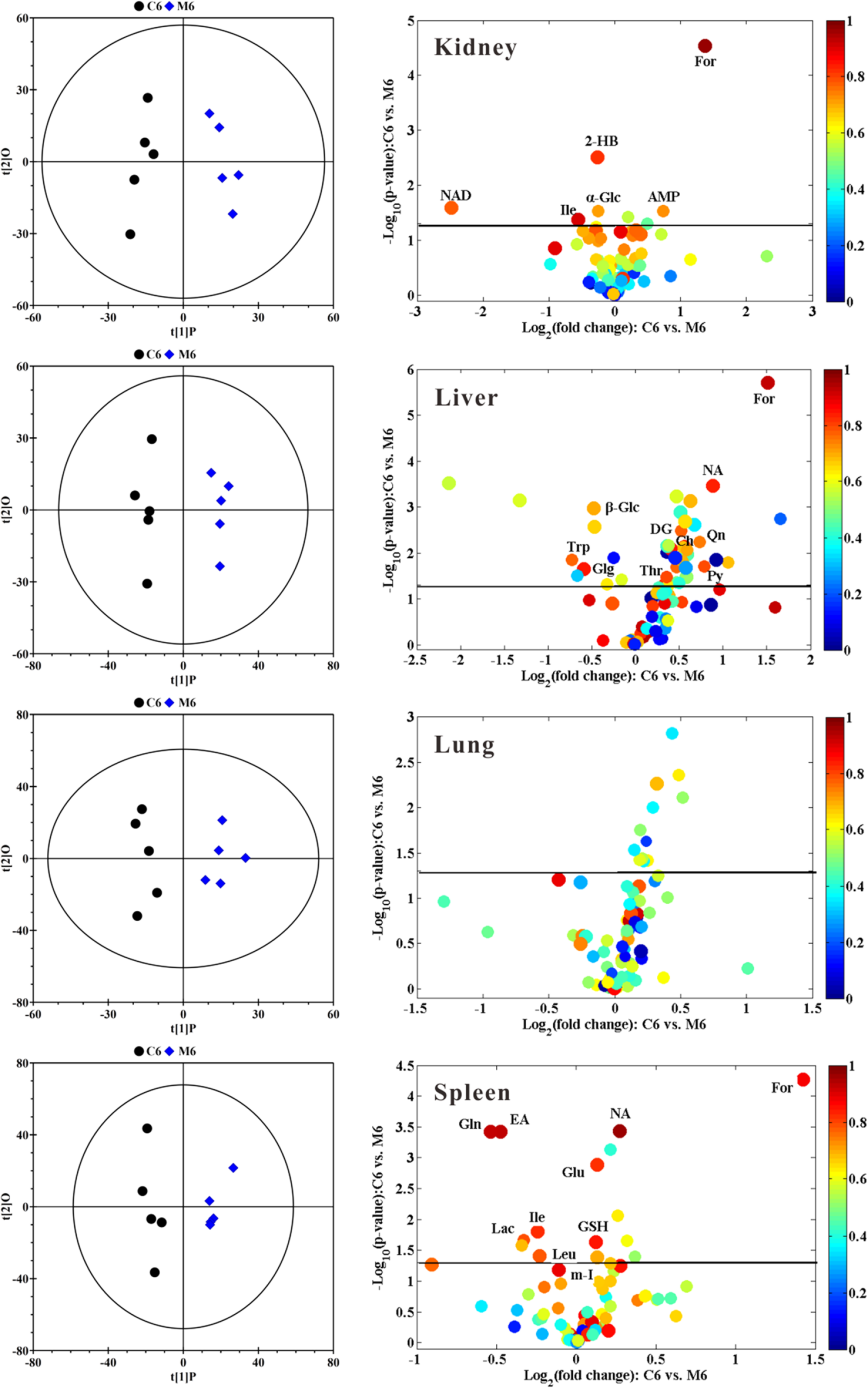
OPLS-DA scores plots (left panels) and corresponding volcano plots (right panels) derived from the ^1^H NMR data of kidney, liver, lung, and spleen obtained from the pairwise groups at 6 h post-administration of mid-size Fe@Si-NPs. C and M represent the control group and the mid-size Fe@Si NPs group (around 20 nm in diameter), respectively; 6 represents 6 h post-treatment. Marked dots in color volcano plots represent metabolites with statistically significant differences. Keys for the assignments are shown in Additional file 1: Table S1

**Fig. 4:**
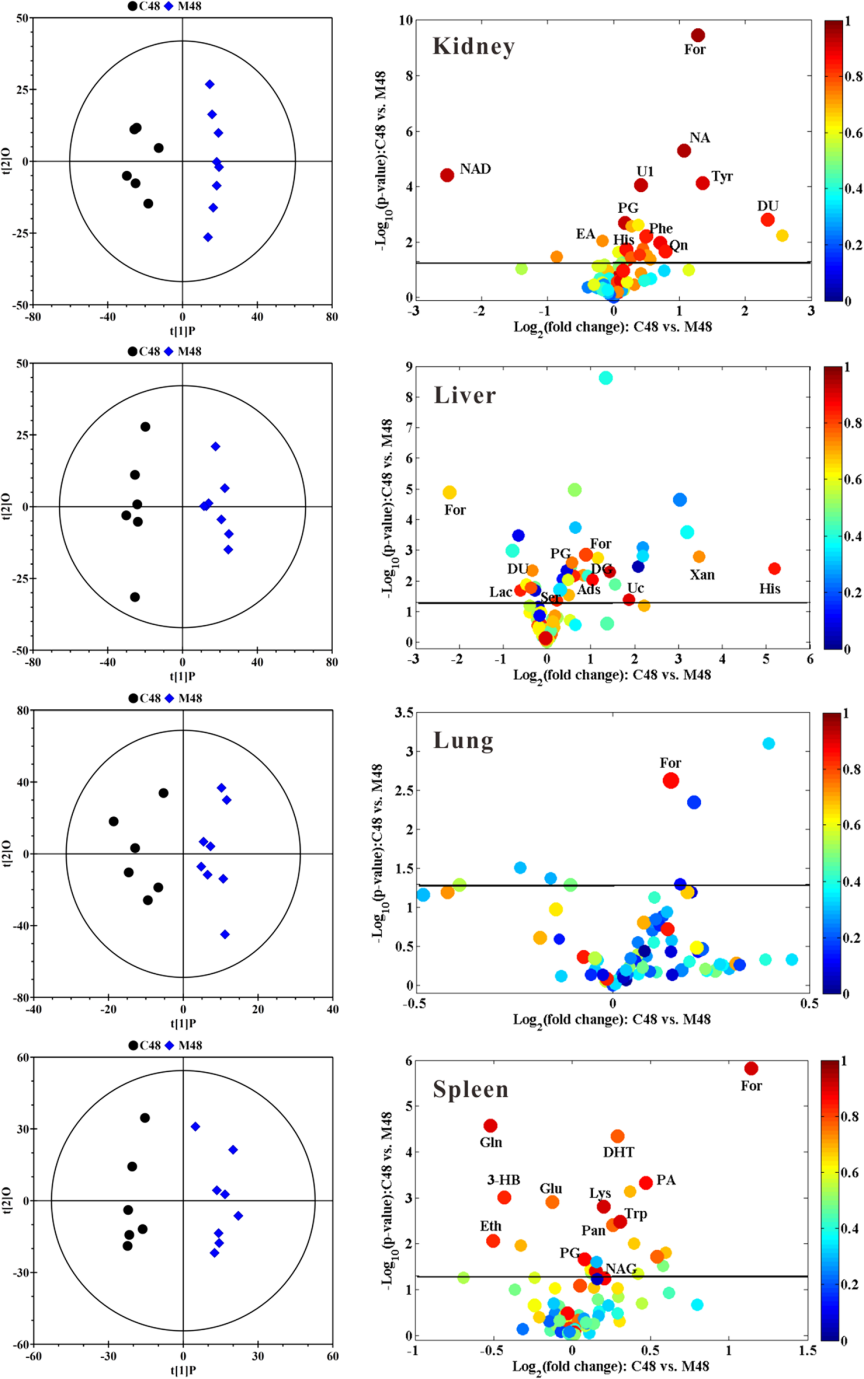
OPLS-DA scores plots (left panel) and corresponding volcano plots (right panels) derived from the ^1^H NMR data of kidney, liver, lung, and spleen obtained from the pairwise groups at 48 h post-administration of mid-size Fe@Si-NPs. C and M represent the control group and the mid-size Fe@Si NPs group (around 20 nm in diameter), respectively, 48 represents 48 h post-treatment. Marked dots in color volcano plots represent metabolites with statistically significant differences. Keys for the assignments are shown in Additional file 1: Table S1

To get the overall metabolic information and examine the intrinsic variation within the respective bio-compartment, supervised OPLS-DA models on the respective NMR data sets were established to identify the metabolic difference between the groups. The OPLS-DA scores plots (left panels in Figs. 3, 4 and Additional file 1: Figures S4–S7) give a significant (P < 0.05) separation between Fe@Si-NPs-treated groups and the corresponding controls, and the volcano plots (right panels in Figs. 3, 4 and Additional file 1: Figures S4–S7) offer an insight into the types of metabolites responsible for the separation. These metabolites provide the biochemical changes in the different bio-compartments following the administration of Fe@Si-NPs. The detailed metabolic information including the VIP values and coefficients from multivariate statistical analysis and the *P*-values from univariate statistical analysis was listed in Additional file 1: Tables S2–S5.

### Metabolomics analysis of kidney metabolome

The overall characteristics of kidney metabolome were that the larger nanoparticle induced the more obvious changes of the metabolites both at 6 h p.d. and 48 h p.d. (Additional file 1: Table S2).

In renal homogenates, an increase of formate and nicotinamide levels and a decrease of NAD level were displayed in near all six treated groups (Additional file 1: Table S2). These metabolites provide a sensitive indicator of their involvement in toxic events induced by Fe@Si-NPs. The increases of 3-methylhistidine, acetate, deoxyguanosine, pyroglutamate, tyrosine and uridine diphosphate glucose levels, and the decrease of isobutyrate level (Additional file 1: Table S2) were, to some extent, correlated with increasing of the size of the nanoparticles, thus implying to be size-dependent. In addition, Fe@Si-NPs also exhibited some size-specific metabolic responses, in which the changes of 3-hydroxybutyrate, alanine, aspartate, cholate, choline, dihydrothymine, ethanol, ethanolamine and glucose were only for the small-size group, deoxyuridine, fumarate, glutamate, histidine, quinone, uridine and urocanate only for the mid-size group, and AMP, glycerol, glycerophosphorylcholine, methylmalonate, pantothenate, threonine, trigonelline and valine only for the large-size group (Additional file 1: Table S2).

### Metabolomics analysis of liver metabolome

The overall characteristics of liver metabolome were similar to those of kidney, namely, the larger nanoparticles induced the more metabolite variations. Liver metabolomic recovery in the three treatment groups at 48 h p.d. could be observed to a certain degree, though not complete, which offers some clues of the biochemical processing of Fe@Si-NPs in the body. However, no metabolite was classified as sensitive indicator. The increases of deoxyuridine, formate, glycogen, histidine, lysine, threonine, urocanate and glucose levels, and the decrease of lactate and tyrosine levels (Additional file 1: Table S3) were, to some extent, correlated with increasing of the size of Fe@Si-NPs. Therefore these metabolites were classified as size-dependent indicators. In addition, Fe@Si-NPs also exhibited size-specific metabolic response, in which the changes of ethanolamine, glycerol, sarcosine, succinate, trigonelline, tryptophan and uridine were only for the small-size group, choline, deoxyguanosine, glutamine, nicotinamide, pyroglutamate, quinone and trimethylamine only for the mid-size group, and alanine, AMP, aspartate, ATP, isobutyrate, NADH, picolinate, valine and xanthine only for the large-size group (Additional file 1: Table S3).

### Metabolomics analysis of lung metabolome

In the lung metabolomes, at 6 h p. d. the small-size of Fe@Si-NPs induced the most variations of metabolites compared with other sizes (Additional file 1: Table S4). At 48 h p. d. the large-size of Fe@Si-NPs induced prominent shift in metabolite profile (Fig. 4). Formate level was elevated in near all treated groups (Additional file 1: Table S4), which made formate as the only sensitive indicator for the lung toxic events induced by Fe@Si-NPs. The increases of *myo*-inositol, propionate and vitamin K1 levels (Additional file 1: Table S4) were, to some extent, correlated with increasing of the size of Fe@Si-NPs. So these metabolites were size-dependent indicators. In addition, Fe@Si-NPs also exhibited size-specific metabolic response in lung metabolome, in which the changes of creatine, glycerophosphorylcholine, glycogen, inosine, lactate and tryptophan were only for the small-size group, ethanol only for the mid-size group, and 1-methylhistidine, adenosine, glutamine, leucine, lysine, *N*-acetylglutamate and pyroglutamate only for the large-size group (Additional file 1: Table S4). The metabolite variations in the small- and mid-size groups were prominently recovered after 48 h p. d. However, the metabolic pathways in large-size group got more seriously affected at 48 h p. d. than at 6 h p. d., which was consistent with the results of biodistribution that showed statistically significant in the large-size group at 48 h p. d.

### Metabolomics analysis of spleen metabolome

The metabolite variations of the spleen were severely affected by Fe@Si-NPs exposure. The overall metabolomic characteristics were that the larger nanoparticles induced the more metabolite variations. The increases of formate, nicotinamide, pyroglutamate and tryptophan levels, and a decrease of glutamine level were displayed in near all six treated groups (Additional file 1: Table S5). Interestingly, isoleucine was decreased at 6 h. p.d. but increased at 48 h p. d. in spleen tissue (Additional file 1: Table S5). These metabolites were defined as sensitive indicators for the spleen toxicity of Fe@Si-NPs. The elevation of aspartate, dihydrothymine, glutathione, *N*-acetylglutamate, pantothenate, and phenylalanine levels, and the reduction of 3-hydroxybutyrate, creatine, ethanol, NAD and serine levels (Additional file 1: Table S5) were, to some extent, correlated with increasing of the size of the Fe@Si-NPs, implying that the changes of these metabolites were size-dependent. In addition, Fe@Si-NPs also exhibited size-specific metabolic response, in which the changes of glycogen and NADP were only for the small-size group, alanine and ethanolamine only for the mid-size group, and acetate, AMP, glycerol, glycerophosphorylcholine, malonate, myo-inositol, quinone, sarcosine, succinate, trimethylamine and glucose only for the large-size group (Additional file 1: Table S5).

## Discussion

### Correlation between nanoparticle sizes, biodistribution, and biological effects of Fe@Si-NPs

The iron content in the kidneys was not significantly affected at 6 h p. d. in the different size groups, while at 48 h p. d., the renal iron contents in the small- and large-size groups displayed a significant increase to 176% and 159% of the control, respectively. Previous research indicated that small IONPs (10 nm) were more likely to be trapped in the liver and cleared from the kidneys [25]. In our study, the increased iron level in kidneys at 48 h p. d. in the small-size group would mean that 10 nm of Fe@Si-NPs were transported to the kidneys for excretion. Another study indicated that the nanoparticles in about 40 nm in diameter are more readily taken up by endothelial cells and induce greater cytotoxicity than the smaller ones [28]. The increased iron level of large-size group in kidneys suggested the increased renal uptake of 40 nm of Fe@Si-NPs at 48 h p. d. The observation that the more metabolites were affected by the large-size Fe@Si-NPs exposure than that of small-size ones in renal metabolome supported to the idea that the increased renal uptake of Fe@Si-NPs (40 nm), rather than increased excretion, accounted for the increased iron content in kidneys. Interestingly, the mid-size group showed no significant accumulation of iron in the kidneys, suggesting that they possibly possess a long blood circulation time and thus are suitable as contrast agents for imaging.

All three sizes of Fe@Si-NPs were rapidly accumulated in spleen and liver at 6 h p. d., and the different sizes did not show fundamental differences. However, as contrast with the small-size group, the iron contents in mid- and large-size groups displayed a significant decrease at 48 h p. d. This discrepancy between iron content (positively correlated to nanoparticle concentration) and biological/toxic effects implied that there are differences in the involved mechanisms, and the size effects play a role in determining the biological fate of Fe@Si-NPs.

Liver clearance involves both reticuloendothelial system cells (Kupffer cells) and non-phagocytic hepatocytes. Particles ingested by Kupffer cells frequently remain within the cells, while particles taken up by hepatocytes can be eliminated by excretion. Most importantly, hepatocytes represent a potential site for toxicity [29]. Therefore, we suppose that small-size Fe@Si-NPs would be more readily ingested by phagocytic cells such as Kupffer cells, and showed a slow turnover; but the mid- and large-size Fe@Si-NPs would be mainly taken up by hepatocytes and showed more toxicity. The results of the metabolomic analysis indirectly support this conclusion, namely, the liver in the small-size group was demonstrated with high iron content (217% of the control, Fig. 1) at 48 h p. d. but without significant side effects on metabolic pathways (Additional file 1: Table S3). In the spleen, a similar discrepancy also existed to that of liver. Therefore we believe that the different sizes of Fe@Si-NPs were taken up by the different cell types, thus leading to the different turnover ratio and the toxic effects.

### Correlation between nanoparticle sizes and NAD depletion

**Fig. 5:**
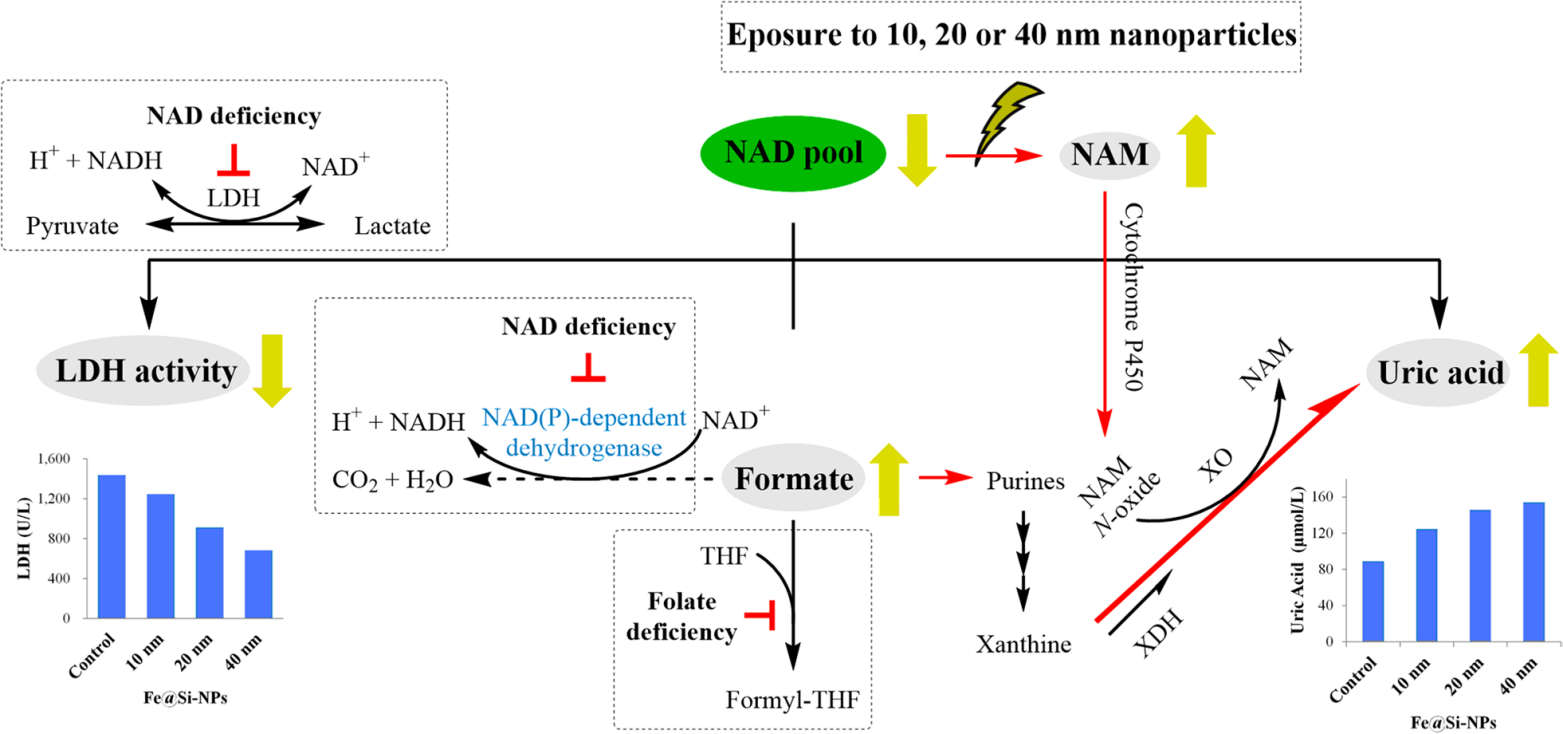
Schematic representation of the proposed mechanism for the in vivo size effects of Fe@Si-NPs on NAD depletion. Green arrows indicate up- or down-regulation; red arrows represent activation; broken arrow indicates less known mechanisms; red T bars represent inhibition. *LDH* lactate dehydrogenase, *NAD* nicotinamide adenine dinucleotide, *NAM* nicotinamide, *NAM N-oxide* nicotinamide *N*-Oxide, *XO* xanthine oxidase, *XDH* xanthine dehydrogenase

LDH is an important enzyme in cellular metabolism, and it is found in almost all of body's cells and known as an enzyme that represents the viability of the cell. It catalyzes the reduction of pyruvate to lactate along with NADH to NAD. In clinical diagnosis, elevations in LDH activity in serum indicate tissue or cellular damage, which can be observed in myocardial infarction, liver disease, severe shock, anemia, muscular dystrophy, malignancies and hypoxia [30, 31]. The reduced LDH activity is usually harmless and can be caused by genetic mutations or other factors that can lower enzyme levels including reducing protein synthesis or increasing protein breakdown in the cell. The coenzyme NAD is necessary for LDH activity. In the absence of NAD, LDH couldn’t exert its catalytic activity. Thus there is a possibility that the coenzyme NAD deficiency in response to Fe@Si-NPs exposure is the cause of this negative correlation between LDH activity and Fe@Si-NPs sizes (Table 1, Fig. 5). This hypothesis was further corroborated by the results of metabolomic analysis, namely Fe@Si-NPs exposure led to a significant decrease of NAD levels and a rise of nicotinamide level (Additional file 1: Tables S2, S3 and S5, Figs. 3, 4 and 5). These two metabolites are reactant and product of NAD-consuming reactions, respectively. ADP-ribosylations, which arise during DNA damage, repair, or recombination, are known to be major NAD-consuming reactions in cell [32]. However, the direct correlation between the properties of nanomaterials, such as size or amino functionalization, and the regulation of NAD-producing or -consuming pathways needs to be further investigated. In addition, intracellular NAD is synthesized de novo from Trp via kynurenine pathway that accounts for about 95% of all tryptophan catabolism. Thus Trp is important for the maintenance of NAD level in body. The alterations of Trp (Additional file 1: Table S5, Fig. 4) in our study suggest that there may be a close correlation between Fe@Si-NPs exposure and NAD synthesis.

One of the most significant metabolic changes was the rise of formate following the administration of Fe@Si-NPs. Formate can be produced from a variety of formate precursors (serine, glycine, tryptophan, histidine, choline, methionine, cholesterol and branched chain fatty acids), and also participates in a number of biosynthetic pathways [33, 34]. Formate can be catalyzed by mitochondrial 10-formyl-tetrahydrofolate synthetase and incorporated into the folate dependent one-carbon pool as 10-formyl-tetrahydrofolate, which provides one-carbon for the synthesis of nucleotides or other biological compounds. This metabolic disposition may be the primary route for formate elimination. As a critical co-factor for formate incorporating into one-carbon pool, folate deficiency would affect the production and turnover of formate. Pigs subjected to a folate deficiency diet exhibited a significant decrease (55%) in the systemic formate clearance [35]. Vitamin B_12_ is at the intersection of the folate-mediated one-carbon pathway and the methionine cycle. The conversion of 5-methyl-tetrahydrofolate back to tetrahydrofolate requires vitamin B_12_. Vitamin B_12_ deficiency can lead to a secondary folate deficiency (methyl-trap hypothesis) and finally the elevated plasma formate levels in rats [36].

Folate and vitamin B_12_ play an important role in supplying active methyl group for the detoxification of xenobiotics. Previous studies have shown that nanoparticles caused dysfunction of the methionine cycle and induced global hypomethylation in HaCaT cell line [37, 38]. In this study, we assume that Fe@Si-NPs exposure resulted in an active methyl group depletion and dysfunction of folate-mediated one-carbon metabolism, and subsequently decreased the assimilation of formate to the folate one-carbon pool, thus causing formate accumulation in tissues (Additional file 1: Tables S2–S5, Figures S4–S7, Figs. 3, 4, 5).

In addition, our knowledge of the catabolic fate of formate is limited. Animals do not possess NAD-dependent formate dehydrogenase that can directly converse formate into CO_2_ [39]. It has been reported that tetrahydrofolate-bound formyl group or formate can be catabolized to CO_2_ by NADP-dependent 10-formyltetrahydro-folate dehydrogenase [40] or catalase [41]. However, the extent to which 10-formyltetrahydro-folate dehydrogenase and catalase play a role in formate removal is uncertain. Thus we cannot exclude the possibility that an unknown NAD/NADP-dependent dehydrogenase exists for catalyzing the oxidation of formate to CO_2_, and the coenzyme NAD deficiency caused by Fe@Si-NPs exposure prevents formate breakdown, and leads to a rise of formate in tissues (Fig. 5).

One of the most noteworthy metabolic characteristics is the positive correlation between serum uric acid level and Fe@Si-NPs sizes. As an important intermediate metabolite in purine catabolism, the regulation of serum uric acid level is a complex process involving hepatic production, renal and gut excretion. Xanthine oxidoreductase (XOR) is the rate limiting enzyme of uric acid synthesis, and its two forms, xanthine dehydrogenase (XDH) and xanthine oxidase (XO), can both convert xanthine to uric acid but via different electron acceptor, in which XDH prefers NAD and XO prefers O_2_ [42]. Under an inflammatory or hypoxic condition, XDH can be converted into oxidase type. Furthermore, nicotinamide can be oxidized by the cytochrome P450 enzyme to nicotinamide *N*-oxide, which can subsequently be catalyzed by xanthine oxidase to transfer O from nicotinamide *N*-oxide to xanthine in the course of uric acid formation [43]. Therefore, we suppose that NAD(P) deficiency would promote the conversion of XDH to XO, and the elevated nicotinamide level would stimulate the production of nicotinamide *N*-oxide. These two aspects may contribute to the increased production of uric acid. The bigger nanoparticles may induce more serious NAD deficiency and produce more nicotinamide, and subsequently more XO and more nicotinamide *N*-oxide, and eventually produce more uric acid. This may be one reason for the positive correlation between level variation of uric acid and Fe@Si-NPs sizes (Table 1, Fig. 5).

In addition, formate is a precursor of purine synthesis. The work by Meiser et al. [44] using ^13^C-methanol to trace the fate of formate in vivo demonstrated that ^13^C can be incorporated into uric acid, a product in purine catabolism, or even faster than that into ATP. An increase of purine synthesis can effectively lower the circulating formate levels. The elevations of formate in most treatment groups were observed in the metabolomic analysis (Figs. 3, 4, 5 and Additional file 1: Figures S4–S7, Tables S2–S5). Therefore, the accumulations of formate would also contribute to the rise of uric acid, as observed in serum biochemical analysis (Table 1, Fig. 5).

### GSH mediated detoxification of Fe@Si-NPs

Glutathione (GSH), a tripeptide made up of three amino acids—glycine, cysteine, and glutamate, is synthesized and metabolized via the γ-glutamyl cycle. GSH conjugation reaction plays a crucial role in detoxification against xenobiotics. The size-dependent increases of GSH levels in spleen (Additional file 1: Table S5, Fig. 3) were associated with the stimulated activities of GSH in response to the exposure of the bigger size Fe@Si-NPs. Consistent with this result, the levels of pyroglutamate, an intermediate in glutathione metabolism, were increased in kidney and spleen tissues in most treatment groups (Additional file 1: Tables S2 and S5, Figures S5, S6 and Figs. 4). In GSH synthesis, both α-ketoglutarate and glutamine are sources of glutamate. Isoleucine is metabolized by branch chain aminotransferase to glutamate via the reaction: isoleucine + α-ketoglutarate → glutamate + α-keto-β-methyvaleric acid. Thus the changes of isoleucine, on the contrary to glutamate, were decreased in all treatment groups at 6 h p. d. but increased at 48 h p. d. in spleen (Additional file 1: Table S5 and Fig. 3). In addition, we noticed that the levels of glutamine were decreased in nearly all treatment groups both at 6 h p. d. and at 48 h p. d. in spleen (Additional file 1: Table S5). The possible reason is that glutamine is not only a precursor to glutamate (catalyzed by glutaminase) but also an important source for de novo purine synthesis. The rise of uric acid, a catabolite of purine metabolism, supports that de novo purine synthesis occurred actively (Table 1). These results suggest that GSH and its related enzymes play an important role in the detoxification of Fe@Si-NPs.

## Conclusions

In this study, we systematically explored the size effects on the in vivo biodistribution and the potential toxicity of amino-functionalized Fe_3_O_4_@SiO_2_ nanoparticles in rats by untargeted metabolomic strategy. Our results indicate that (i) different sizes of Fe@Si-NPs induced different biological process and activities including biodistribution, biological effects and underlying mechanisms; (ii) LDH, formate, uric acid, and GSH related metabolites were suggested as sensitive indicators for the size-dependent toxic effects of Fe@Si-NPs; (iii) NAD regulated LDH activity which was negatively correlated with Fe@Si-NPs sizes; (iv) Fe@Si-NPs induced the dysfunction of folate-mediated one-carbon metabolism, thus causing the formate accumulation in tissues; (v) The bigger size of Fe@Si-NPs induce more xanthine oxidoreductase activities and produce more uric acid. (vi) GSH is important for the detoxification of Fe@Si-NPs. Whether these toxic hypotheses are of general significance will need further validation in a wide range of nanoparticles. However, our findings provide the ground for the size selection and optimization, further contributing to the biological applications of Fe@Si-NPs.

## Supplementary information

**Additional file 1: Figure S1.** TEM image of water-dispersible 10 (a), 20 (b) and 40 (c) nm of Core-shell Structured Fe_3_O_4_@SiO_2_–NH_2_ nanoparticles. **Figure S2.** Effect of Fe@Si-NPs administration on tissue/body weight ratio (%). **Figure S3.** Photomicrographs of representative sections of the lung (A, B, C and D), liver (E, F, G and H), spleen (I, J, K and L) and kidney (M, N, O and P) from control (A, E, I and M), small-size (B, F, J and N), mid-size (C, G, K and O), and large-size (D, H, L and P) Fe@Si-NPs treated rats at 48 h p. d. **Figure S4.** OPLS-DA scores plots (left panel) and corresponding volcano plots (right panels) derived from the ^1^H NMR data of kidneys, liver, lung, and spleen obtained from the pairwise groups at 6 h post-administration of small-size Fe@Si-NPs. **Figure S5.** OPLS-DA scores plots (left panels) and corresponding volcano plots (right panels) derived from the ^1^H NMR data of kidney, liver, lung, and spleen obtained from the pairwise groups at 6 h post-administration of large-size Fe@Si-NPs. **Figure S6.** OPLS-DA scores plots (left panels) and corresponding volcano plots (right panels) derived from the ^1^H NMR data of kidney, liver, lung, and spleen obtained from the pairwise groups at 48 h post-administration of small-size Fe@Si-NPs. **Figure S7.** OPLS-DA scores plots (left panels) derived from the ^1^H NMR data of kidney, liver, lung, and spleen and corresponding volcano plots (right panels) obtained from the pairwise groups at 48 h post-administration of large-size Fe@Si-NPs. **Table S1.** The metabolites identified from the NMR spectra of tissue samples. **Table S2.** Summary of metabolic variations in kidney extracts induced by Fe@Si-NPs between different pairwise groups. **Table S3.** Summary of metabolic variations in liver extracts induced by Fe@Si-NPs between different pairwise groups. **Table S4.** Summary of metabolic variations in lung extracts induced by Fe@Si-NPs between different pairwise groups. **Table S5.** Summary of metabolic variations in spleen extracts induced by Fe@Si-NPs between different pairwise groups.

## Data Availability

The datasets used and/or analyzed during the current study are available from the corresponding author on reasonable request.
