# Supplementary material for: Size-dependent tissue-specific biological effects of core–shell structured Fe3O4@SiO2–NH2 nanoparticles
Source: J Nanobiotechnology. 2019 Dec 23;17:124. doi: 10.1186/s12951-019-0561-4 (PMC6929447; doi:10.1186/s12951-019-0561-4)
Supplement: Supplementary file 1 — Additional file 1: Figure S1. TEM image of water-dispersible 10 (a), 20 (b) and 40 (c) nm of Core-shell Structured Fe3O4@SiO2–NH2 nanoparticles. Figure S2. Effect of Fe@Si-NPs administration on tissue/body weight ratio (%). Figure S3. Photomicrographs of representative sections of the lung (A, B, C and D), liver (E, F, G and H), spleen (I, J, K and L) and kidney (M, N, O and P) from control (A, E, I and M), small-size (B, F, J and N), mid-size (C, G, K and O), and large-size (D, H, L and P) Fe@Si-NPs treated rats at 48 h p. d. Figure S4. OPLS-DA scores plots (left panel) and corresponding volcano plots (right panels) derived from the 1H NMR data of kidneys, liver, lung, and spleen obtained from the pairwise groups at 6 h post-administration of small-size Fe@Si-NPs. Figure S5. OPLS-DA scores plots (left panels) and corresponding volcano plots (right panels) derived from the 1H NMR data of kidney, liver, lung, and spleen obtained from the pairwise groups at 6 h post-administration of large-size Fe@Si-NPs. Figure S6. OPLS-DA scores plots (left panels) and corresponding volcano plots (right panels) derived from the 1H NMR data of kidney, liver, lung, and spleen obtained from the pairwise groups at 48 h post-administration of small-size Fe@Si-NPs. Figure S7. OPLS-DA scores plots (left panels) derived from the 1H NMR data of kidney, liver, lung, and spleen and corresponding volcano plots (right panels) obtained from the pairwise groups at 48 h post-administration of large-size Fe@Si-NPs. Table S1. The metabolites identified from the NMR spectra of tissue samples. Table S2. Summary of metabolic variations in kidney extracts induced by Fe@Si-NPs between different pairwise groups. Table S3. Summary of metabolic variations in liver extracts induced by Fe@Si-NPs between different pairwise groups. Table S4. Summary of metabolic variations in lung extracts induced by Fe@Si-NPs between different pairwise groups. Table S5. Summary of metabolic variations in spleen extracts induced [file 12951_2019_561_MOESM1_ESM.doc]

Additional file 1

Figure S1. TEM image of water-dispersible 10 (a), 20 (b) and 40 (c) nm of core-shell structured Fe_3_O_4_@SiO_2_-NH_2_ nanoparticles.

Figure S2. Effect of Fe@Si-NPs administration on tissue/body weight ratio (%). Keys: C, control group; S, small-size group; M, mid-size group; L, large-size group; 6, 6 h post-dose; 48, 48 h post-dose. No statistically significant difference (*p* values more than 0.05) in the ratio of the tissue weight to the total body weight of rats between all Fe@Si-NPs-treated groups and the corresponding controls.


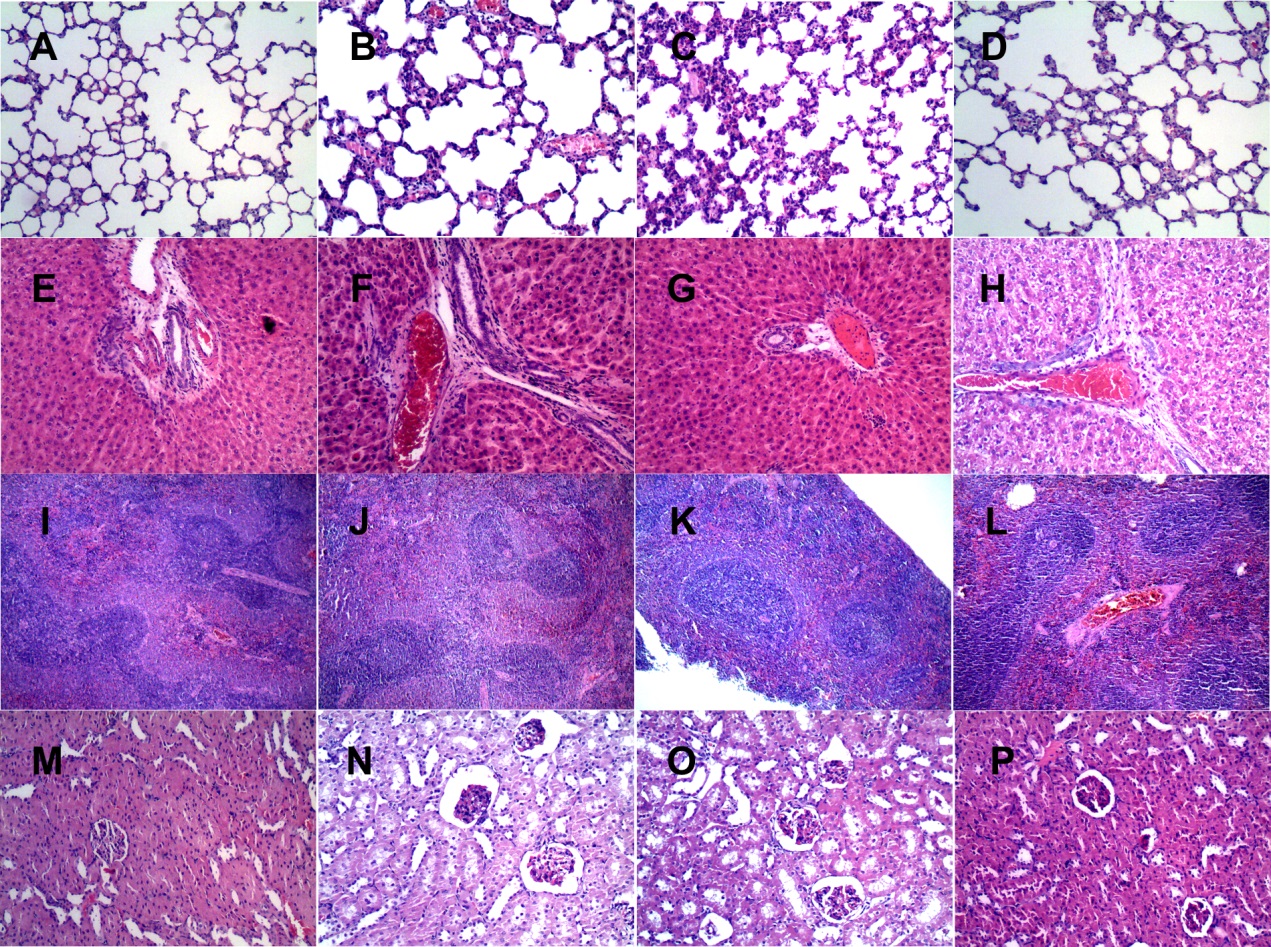


Figure S3. Photomicrographs of representative sections of the lung (A, B, C and D), liver (E, F, G and H), spleen (I, J, K and L) and kidney (M, N, O and P) from the controls (A, E, I and M), small-size (B, F, J and N), mid-size (C, G, K and O), and large-size (D, H, L and P) Fe@Si-NPs treated rats at 48 h p. d.. The tissue sections were stained with hematoxylin-eosin and observed under a 100× [microscope](javascript:void(0)).

**
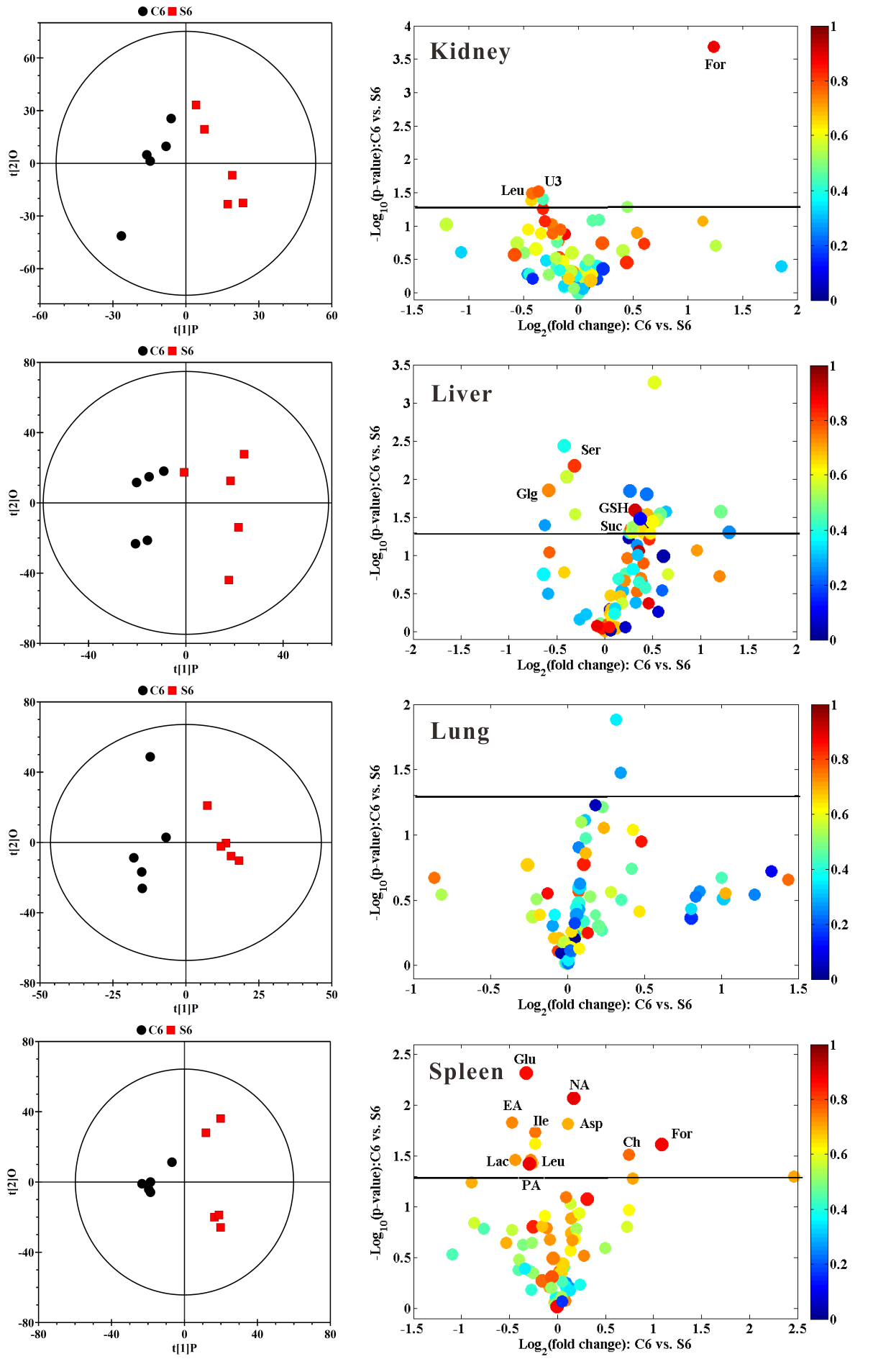
**

Figure S4. OPLS-DA scores plots (left panel) and corresponding volcano plots (right panels) derived from the ^1^H NMR data of kidneys, liver, lung, and spleen obtained from the pairwise groups at 6 hours post-administration of small-size Fe@Si-NPs. C and M represent the control group and the small-size Fe@Si NPs group (around 10 nm in diameter), respectively; 6 represents 6 hours post-treatment. Marked dots in color volcano plots represent metabolites with statistically significant differences. Keys for the assignments are shown in Table S1.

**
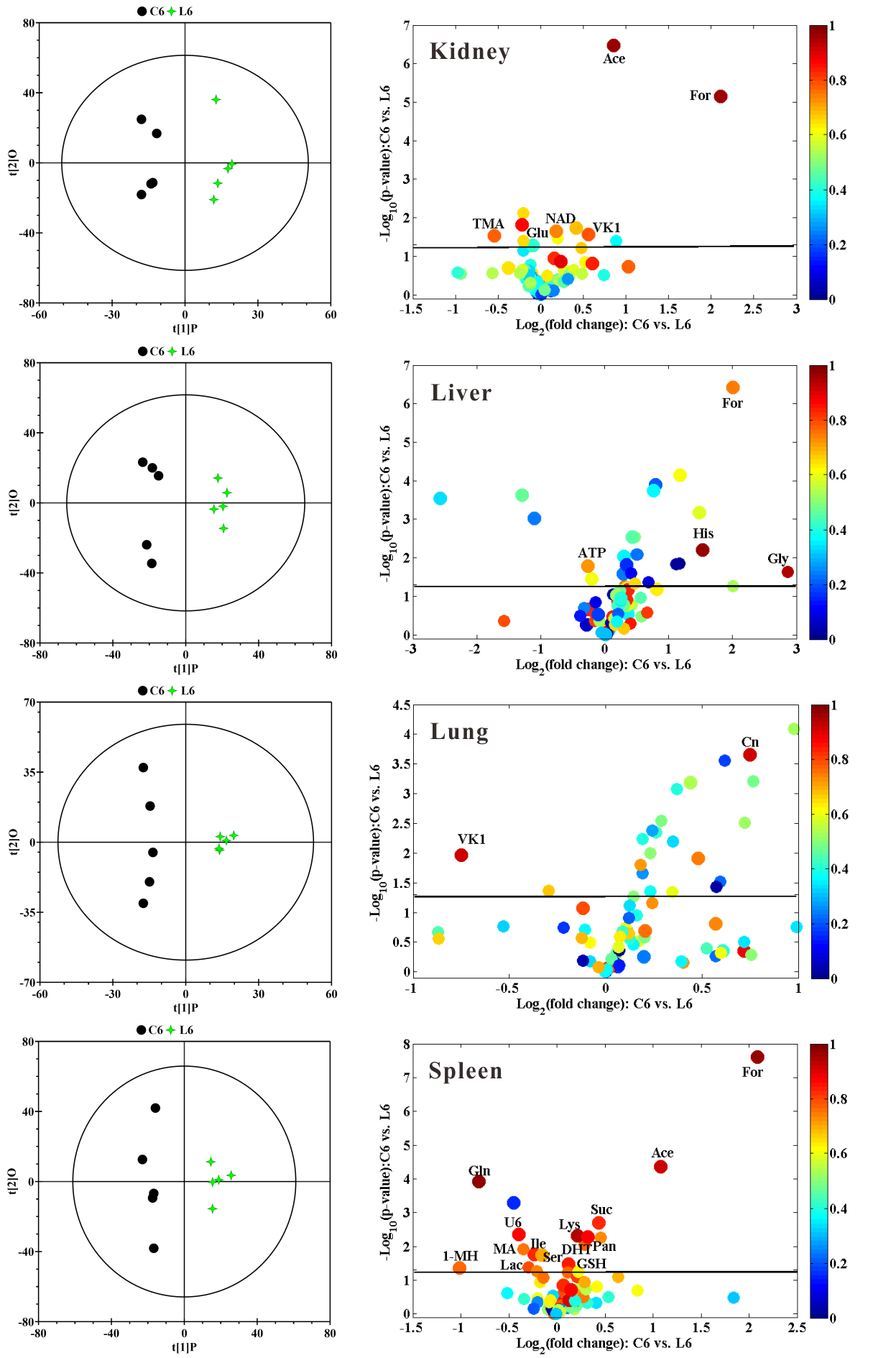
**

Figure S5. OPLS-DA scores plots (left panels) and corresponding volcano plots (right panels) derived from the ^1^H NMR data of kidney, liver, lung, and spleen obtained from the pairwise groups at 6 hour post-administration of large-size Fe@Si-NPs. C and M represent the control group and the large-size Fe@Si NPs group (around 40 nm in diameter), respectively; 6 represents 6 hours post-treatment. Marked dots in color volcano plots represent metabolites with statistically significant differences. Keys for the assignments are shown in Table S1.


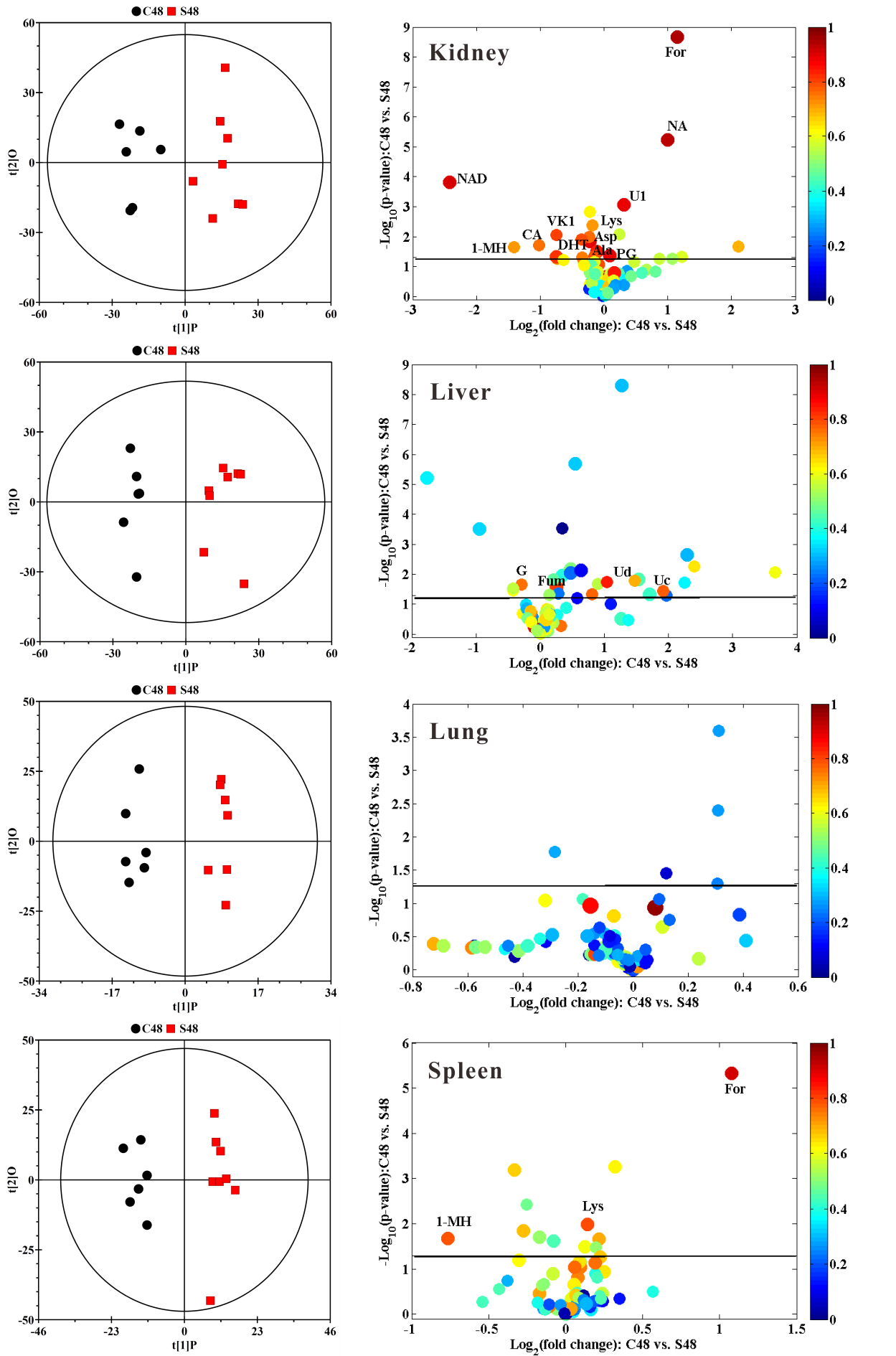


Figure S6. OPLS-DA scores plots (left panels) and corresponding volcano plots (right panels) derived from the ^1^H NMR data of kidney, liver, lung, and spleen obtained from the pairwise groups at 48 h post-administration of small-size Fe@Si-NPs. C and M represent the control group and the large-size Fe@Si NPs group (around 10 nm in diameter), respectively; 48 represents 48 hours post-treatment. Marked dots in color volcano plots represent metabolites with statistically significant differences. Keys for the assignments are shown in Table S1.

**
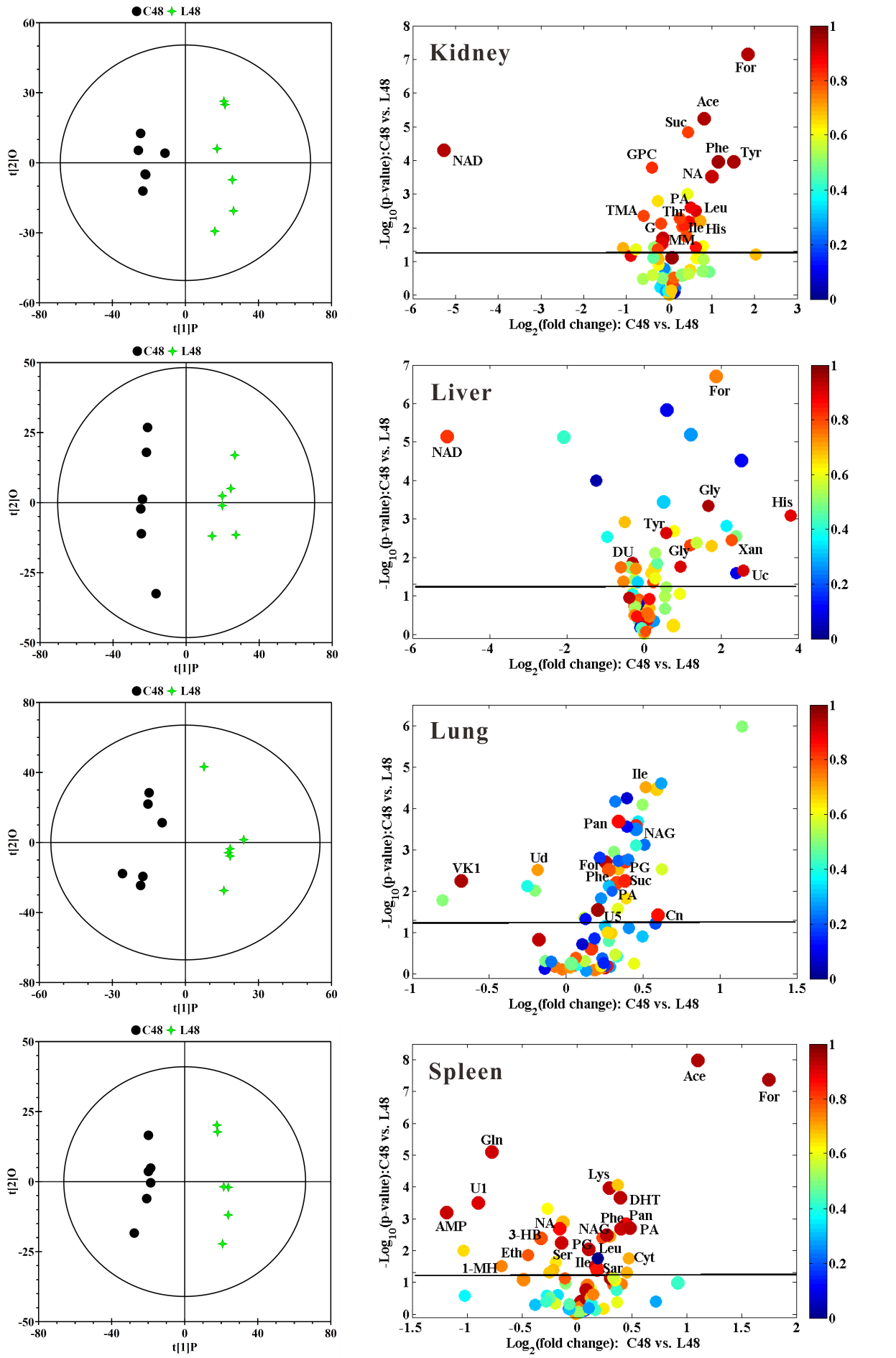
**

Figure S7. OPLS-DA scores plots (left panels) derived from the ^1^H NMR data of kidney, liver, lung, and spleen and corresponding volcano plots (right panels) obtained from the pairwise groups at 48 h post-administration of large-size Fe@Si-NPs. C and M represent the control group and the large-size Fe@Si NPs group (around 40 nm in diameter), respectively; 48 represents 48 hours post-treatment. Marked dots in color volcano plots represent metabolites with statistically significant differences. Keys for the assignments are shown in Table S1.

**Table S1** The metabolites identified from the NMR spectra of tissue samples

| **Metabolites** | **Abbr.** | **^1^H Chemical Shift (multiplicity)** | **Sample** |
| --- | --- | --- | --- |
| 1-Methylhistidine | 1-MH | 7.06(s^a^); 7.78(s) | Ki^b^,Li,Lu,Sp |
| 2-Hydroxyisobutyrate | 2-HB | 1.37(s) | Ki,Li |
| 2-Ketobutyrate | 2-KB | 1.08(t) | Li |
| 3-Hydroxybutyrate | 3-HB | 1.20(d); 2.31(m); 2.41(m); 4.16(m) | Ki,Li,Lu,Sp |
| 3-Methylhistidine | 3-MH | 7.05(s), 7.68(s) | Ki,Li,Lu,Sp |
| Acetate | Ace | 1.92(s) | Ki,Li,Lu,Sp |
| Acetone | Act | 2.23(s) | Ki |
| Adenine | Ad | 8.12(s) | Ki,Li,Lu,Sp |
| Adenosine | Ads | 4.30(dd); 4.44(dd); 6.07(d); 8.26(s); 8.34(s) | Ki,Li,Lu,Sp |
| Adenosine diphosphate | ADP | 6.15(d);8.58(s) | Ki,Li,Lu,Sp |
| Adenosine monophosphate | AMP | 4.03(m); 4.37(m); 4.51(m); 6.14(d); 8.26(s); 8.62(s) | Ki,Li,Lu,Sp |
| Adenosine triphosphate | ATP | 8.56(s); 8.56(s) | Ki,Li,Lu,Sp |
| Alanine | Ala | 1.48(d); 3.78(q) | Ki,Li,Lu,Sp |
| Aspartate | Asp | 2.67(dd); 2.82(dd); 3.90(dd) | Ki,Li,Lu,Sp |
| Betaine | Bet | 3.27(s); 3.89(s) | Ki,Li,Lu,Sp |
| Cholate | CA | 0.72(m) | Ki,Li,Lu,Sp |
| Choline | Ch | 3.20(s); 3.52(m); 4.07(m) | Ki,Li,Lu,Sp |
| Creatine | Cr | 3.04(s); 3.93(s) | Ki,Li,Lu,Sp |
| Creatinine | Cn | 3.05(s); 4.06(s) | Lu |
| Cytidine | Cyt | 5.89(d); 6.06(d); 7.84(d) | Ki,Li,Lu,Sp |
| Deoxyguanosine | DG | 7.99(s) | Ki,Li,Lu,Sp |
| Deoxyuridine | DU | 6.27(t) | Ki,Li,Lu,Sp |
| Dihydrothymine | DHT | 1.07(d) | Ki,Lu,Sp |
| Dimethylamine | DMA | 2.72(s) | Ki,Lu,Li |
| Dimethylglycine | DMG | 2.93(s); 3.73(s) | Ki,Li,Lu,Sp |
| Ethanol | Eth | 1.19(t); 3.66(q) | Ki,Li,Lu,Sp |
| Ethanolamine | EA | 3.14(t); 3.83(t) | Ki,Li,Lu,Sp |
| Formate | For | 8.46(s) | Ki,Li,Lu,Sp |
| Fumarate | Fum | 6.52(s) | Ki,Li,Lu,Sp |
| Glutamate | Glu | 2.05(m); 2.13(m); 2.35(m); 3.78(t) | Ki,Li,Lu,Sp |
| Glutamine | Gln | 2.14(m); 2.46(m); 3.78(t) | Ki,Li,Lu,Sp |
| Glutathione | GSH | 2.17(m); 2.55(m); 2.98(dd); 3.78(m); 4.20(q) | Ki,Li,Lu,Sp |
| Glycerol | G | 3.57(m); 3.62(m); 3.79(m) | Ki,Li,Lu,Sp |
| Glycerophosphorylcholine | GPC | 3.23(s); 4.33(m) | Ki,Li,Lu,Sp |
| Glycine | Gly | 3.56(s) | Ki,Li,Lu,Sp |
| Glycogen | Glg | 3.66(m);3.86(m);3.99(m); 5.40(m) | Ki,Li,Lu,Sp |
| Histidine | His | 3.14(m); 4.00(m); 7.08(s), 7.83(s) | Ki,Li |
| Inosine | Ino | 4.28(dd); 4.44(t); 6.09(d); 8.22(s); 8.34(s) | Ki,Li,Lu,Sp |
| Isobutyrate | IB | 1.07(d) | Ki,Li |
| Isoleucine | Ile | 0.94(t); 1.01(d) | Ki,Li,Lu,Sp |
| Lactate | Lac | 1.33(d); 4.11(q) | Ki,Li,Lu,Sp |
| Leucine | Leu | 0.96(t); 1.70(m) | Ki,Li,Lu,Sp |
| Lysine | Lys | 1.73(m); 1.88(m); 3.03(t) | Ki,Li,Lu,Sp |
| Malonate | MA | 3.11(s) | Ki,Li,Lu,Sp |
| Methionine | Met | 2.14(s); 2.16(m); 2.64(t); 3.86(t) | Ki |
| Methylamine | MA | 2.61(s) | Ki |
| Methylmalonate | MM | 1.24(d) | Ki |
| myo-Inositol | m-I | 3.28(t); 3.54(dd); 3.63(t); 4.07(t) | Ki,Li,Lu,Sp |
| N-Acetylglutamate | NAG | 1.86(m); 2.04(s); 2.25(t) | Li,Lu,Sp |
| Nicotinamide | NA | 7.59(dd); 8.24(dd); 8.94(s) | Ki,Li,Lu,Sp |
| Nicotinamide adenine dinucleotide | NAD | 4.49(dd); 4.55(m); 6.04(d); 6.09(d); 8.18(s);8.21(m); 8.43(s); 8.84(d); 9.15(d); 9.34(s) | Ki,Li,Lu,Sp |
| Nicotinamide adenine dinucleotide phosphate | NADP | 8.83(d); 9.11(d); 9.29(s) | Li, Ki,Sp |
| N-stearoylsphingosine | NSP | 5.55(d); 5.77(m) | Lu,Sp |
| Pantothenate | Pan | 0.88(s); 0.92(s) | Ki,Li,Lu,Sp |
| Phenylalanine | Phe | 7.33(d); 7.38(t); 7.43(m) | Ki,Li,Lu,Sp |
| Phosphocholine | PC | 3.22(s); 3.59(m); 4.17(m) | Ki,Li,Lu,Sp |
| Picolinate | PA | 7.51(d); 7.92(m); 8.55(d) | Li |
| Propionate | Prop | 1.06(t); 2.18(q) | Ki,Lu,Sp |
| Pterin | Pr | 8.59(s) | Li,Lu,Sp |
| Pyroglutamate | PG | 2.05(m); 2.39(d); 2.51(m); 4.18(dd) | Ki,Li,Lu,Sp |
| Pyruvate | Py | 2.37(s) | Li |
| Quinone | Qn | 6.80(s) | Ki,Li,Lu,Sp |
| Sarcosine | Sar | 2.74(s); 3.60(s) | Ki,Li,Lu,Sp |
| Serine | Ser | 3.83(m); 3.96(m) | Ki,Li,Lu,Sp |
| Sphingosine | Sph | 5.52(dd);5.72(m) | Lu,Sp |
| Succinate | Suc | 2.41(s) | Ki,Li,Lu,Sp |
| Threonine | Thr | 1.33(d); 3.59(d); 4.25(m) | Ki,Li,Lu,Sp |
| Trigonelline | Tri | 4.44(s); 8.08(t); 8.84(dd); 9.13(s) | Ki,Li,Lu |
| Trimethylamine | TMA | 2.88(s) | Ki,Li,Lu,Sp |
| Trimethylamine N-oxide | TMAO | 3.30(s) | Li,Lu,Sp |
| Tryptophan | Trp | 7.19(m); 7.28(m); 7.31(s); 7.53(d); 7.74(d) | Ki,Li,Lu,Sp |
| Tyrosine | Tyr | 6.89(d); 7.19(d) | Ki,Li,Lu,Sp |
| Unknown-1 | U1 | 8.53(m) | Ki,Li,Lu,Sp |
| Unknown-2 | U2 | 8.37(2) | Ki,Li,Lu |
| Unknown-3 | U3 | 6.71(dd) | Ki,Li,Lu |
| Unknown-4 | U4 | 6.21(dd) | Ki,Li,Sp |
| Unknown-5 | U5 | 1.81(s) | Ki,Li,Lu,Sp |
| Unknown-6 | U6 | 3.10(s) | Ki,Li,Sp |
| Unknown-8 | U8 | 4.67(s) | Li,Lu |
| Unknown-9 | U9 | 5.52(dd) | Li |
| Unknown-10 | U10 | 5.68(d) | Li,Sp |
| Unknown-11 | U11 | 5.87(dd) | Li,Sp |
| Unknown-12 | U12 | 5.82(dd) | Ki,Li,Lu,Sp |
| Unknown-13 | U13 | 5.98(m) | Lu |
| Uracil | Ura | 5.80(d); 7.53(d) | Ki |
| Uridine | Ud | 5.90(d); 5.91(d); 7.87(d) | Ki,Li,Lu,Sp |
| Uridine diphosphate glucose | UDG | 5.61(dd); 5.98(m); 7.96(d) | Ki,Li,Lu,Sp |
| Urocanate | Uc | 6.39(d); 7.28(d); 7.37(s); 7.82(s) | Ki,Li,Lu,Sp |
| Valine | Val | 0.99(d); 1.04(d) | Ki,Li,Lu,Sp |
| Vitamine K1 | VK1 | 0.78(s);0.89(s) | Ki,Li,Sp |
| Vitamine K2 | VK2 | 0.80(s) | Li |
| Vitamine K3 | VK3 | 0.83(s) | Li |
| Xanthine | Xan | 7.93(s) | Ki,Li,Lu,Sp |
| α-Glucose | α-Glc | 3.42(t); 3.54(dd); 3.71(t); 3.74(m); 3.84(m); 5.24(d) | Ki,Li,Lu,Sp |
| β-Glucose | β-Glc | 3.25(dd); 3.41(t); 3.46(m); 3.49(t); 3.72(dd); 3.90(dd); 4.65(d) | Ki,Li,Lu,Sp |

^a^ Multiplicity: m, multiplet; br, broad; s, singlet; d, doublet; t, triplet; q, quartet; dd, doublet of doublets.

^b^ Ki, kidneys; Li, liver; Lu, lung; Sp, spleen

**Table S2** Summary of metabolic variations in kidney extracts induced by Fe@Si-NPs between different pairwise groups

| **Metabolites** | **C6-S6** | | | **C6-M6** | | | **C6-L6** | | | **C48-S48** | | | **C48-M48** | | | **C48-L48** | | |
| --- | --- | --- | --- | --- | --- | --- | --- | --- | --- | --- | --- | --- | --- | --- | --- | --- | --- | --- |
|  | **VIP**^a^ | **R**^b^ | **p**^c^ | **VIP** | **R** | **p** | **VIP** | **R** | **p** | **VIP** | **R** | **p** | **VIP** | **R** | **p** | **VIP** | **R** | **p** |
| 1-Methylhistidine | /^d^ | / | / | / | / | / | / | / | / | 1.34 | / | 0.02 | / | / | / | / | / | / |
| 2-Hydroxyisobutyrate | / | / | / | 1.65 | -0.83 | 0.00 | / | / | / | 1.64 | -0.90 | / | / | / | / | / | / | / |
| 3-Hydroxybutyrate | 1.55 | -0.75 | / | / | / | / | / | / | / | 1.38 | -0.75 | 0.01 | / | / | / | / | / | / |
| 3-Methylhistidine | / | / | / | / | / | / | / | / | / | / | / | / | 1.40 | 0.77 | / | 1.34 | 0.79 | 0.01 |
| Acetate | / | / | / | / | / | / | 2.08 | 0.97 | 0.00 | / | / | / | 1.53 | 0.83 | 0.02 | 1.55 | 0.95 | 0.00 |
| Adenine | 1.68 | 0.82 | / | / | / | / | / | / | / | / | / | / | / | / | / | 1.34 | 0.82 | / |
| Adenosine diphosphate | / | / | / | / | / | / | / | / | / | / | / | / | / | / | / | / | / | / |
| Adenosine monophosphate | / | / | / | 1.47 | / | 0.03 | / | / | / | / | / | / | / | / | / | 1.46 | -0.90 | / |
| Alanine | / | / | / | / | / | / | / | / | / | 1.58 | -0.85 | 0.03 | / | / | / | / | / | / |
| Aspartate | 1.60 | -0.80 | / | / | / | / | / | / | / | 1.42 | -0.77 | 0.01 | / | / | / | / | / | / |
| Cholate | / | / | / | / | / | / | / | / | / | 1.43 | -0.76 | 0.02 | / | / | / | / | / | / |
| Choline | / | / | / | / | / | / | / | / | / | 1.49 | -0.79 | 0.05 | / | / | / | / | / | / |
| Deoxyguanosine | / | / | / | / | / | / | / | / | / | / | / | / | 1.55 | 0.86 | 0.02 | 1.42 | 0.82 | / |
| Deoxyuridine | / | / | / | / | / | / | / | / | / | 1.42 | / | 0.02 | 1.61 | 0.84 | / | / | / | / |
| Dihydrothymine | 1.67 | -0.84 | / | / | / | / | / | / | / | 1.47 | -0.80 | 0.01 | / | / | / | / | / | / |
| Ethanol | 1.62 | -0.79 | / | 1.82 | -0.90 | / | / | / | / | 1.51 | -0.83 | 0.05 | 1.39 | / | 0.03 | / | / | / |
| Ethanolamine | 1.74 | -0.87 | / | / | / | / | 1.43 | / | 0.01 | / | / | / | 1.33 | / | 0.01 | / | / | / |
| Formate | 1.77 | 0.89 | 0.00 | 1.94 | 0.98 | 0.00 | 2.07 | 0.97 | 0.00 | 1.74 | 0.95 | 0.00 | 1.75 | 0.97 | 0.00 | 1.54 | 0.95 | 0.00 |
| Fumarate | / | / | / | 1.73 | 0.81 | / | / | / | / | / | / | / | 1.63 | 0.88 | / | / | / | / |
| Glutamate | / | / | / | 1.77 | 0.89 | / | / | / | / | / | / | / | / | / | / | / | / | / |
| Glutamine | / | / | / | / | / | / | 1.87 | -0.87 | 0.02 | 1.64 | 0.89 | / | 1.57 | -0.83 | / | 1.58 | -0.93 | 0.02 |
| Glutathione | / | / | / | / | / | / | 1.63 | 0.76 |  | 1.65 | -0.80 | / | 1.69 | 0.93 | / | 1.54 | -0.90 | 0.03 |
| Glycerol | / | / | / | / | / | / | / | / | / | / | / | / | / | / | / | 1.32 | -0.81 | 0.01 |
| Glycerophosphorylcholine | / | / | / | / | / | / | / | / | / | / | / | / | / | / | / | 1.37 | -0.83 | 0.00 |
| Histidine | / | / | / | / | / | / | / | / | / | / | / | / | 1.47 | 0.82 | 0.01 | / | / | / |
| Isobutyrate | 1.61 | -0.80 |  | 1.57 | -0.79 | / | / | / | / | 1.41 | -0.77 | / | / | / | / | / | / | / |
| Isoleucine | 1.35 | / | 0.04 | 1.83 | -0.91 | 0.04 | / | / | / | / | / | / | / | / | / | 1.43 | 0.88 | 0.01 |
| Leucine | 1.57 | -0.76 | 0.03 | / | / | / | / | / | / | / | / | / | / | / | / | 1.48 | 0.91 | 0.00 |
| Lysine | / | / | / | / | / | / | / | / | / | 1.34 | / | 0.00 | / | / | / | / | / | / |
| Methylmalonate | / | / | / | / | / | / | 1.46 | / | 0.03 | / | / | / | / | / | / | 1.31 | 0.80 | 0.01 |
| Nicotinamide | 1.66 | 0.83 | / | / | / | / | 1.81 | 0.85 |  | 1.72 | 0.94 | 0.00 | 1.73 | 0.95 | 0.00 | 1.52 | 0.93 | 0.00 |
| NAD | / | / | / | 1.56 | -0.78 | 0.03 | 1.87 | -0.87 | 0.01 | 1.66 | -0.91 | 0.00 | 1.68 | -0.93 | 0.00 | 1.55 | -0.95 | 0.00 |
| Pantothenate | / | / | / | / | / | / | 1.66 | / | 0.02 | / | / | / | / | / | / | 1.37 | 0.84 | 0.01 |
| Phenylalanine | 1.66 | -0.84 | / | / | / | / | / | / | / | / | / | / | 1.58 | 0.84 | 0.01 | 1.57 | 0.97 | 0.00 |
| Propionate | / | / | / | 1.70 | 0.82 | / | / | / | / | / | / | / | 1.51 | 0.82 | / | 1.44 | 0.88 | 0.00 |
| Pyroglutamate | / | / | / | / | / | / | 1.79 | 0.83 |  | 1.65 | 0.89 | 0.04 | 1.68 | 0.93 | 0.00 | 1.58 | 0.97 | / |
| Quinone | / | / | / | 1.59 | 0.77 | / | / | / | / | / | / | / | 1.64 | 0.89 | 0.01 | 1.44 | 0.85 | 0.04 |
| Succinate | / | / | / | / | / | / | 1.51 | / | 0.02 | 1.57 | 0.85 | / | / | / | 0.00 | 1.43 | 0.81 | 0.00 |
| Threonine | / | / | / | / | / | / | / | / | / | / | / | / | / | / | / | 1.37 | 0.81 | 0.01 |
| Trigonelline | / | / | / | / | / | / | 1.71 | 0.78 | / | / | / | / | / | / | / | / | / | / |
| Trimethylamine | 1.53 | -0.77 | / | / | / | / | 1.72 | -0.78 | 0.03 | / | / | / | / | / | / | 1.38 | -0.81 | 0.00 |
| Tyrosine | / | / | / | / | / | / | / | / | / | / | / | / | 1.67 | 0.91 | 0.00 | 1.56 | 0.96 | 0.00 |
| Unknown-1 | 1.57 | 0.79 | / | 1.54 | 0.77 | / | 1.92 | 0.90 | / | 1.65 | 0.89 | / | 1.68 | 0.93 | / | 1.36 | 0.80 | / |
| Unknown-12 | / | / | / | / | / | / | / | / | / | / | / | / | 1.65 | 0.90 | / | 1.35 | -0.81 | / |
| Unknown-3 | 1.60 | -0.79 | / | / | / | / | / | / | / | / | / | / | / | / | / | / | / | / |
| Unknown-5 | / | / | / | / | / | / | / | / | / | / | / | / | 1.58 | 0.85 | / | / | / | / |
| Uridine | / | / | / | / | / | / | / | / | / | / | / | / | 1.41 | 0.78 | 0.00 | / | / | / |
| Uridine diphosphate glucose | / | / | / | / | / | / | / | / | / | / | / | / | 1.55 | 0.86 | / | 1.39 | 0.78 | / |
| Urocanate | / | / | / | / | / | / | / | / | / | / | / | / | 1.49 | 0.82 | / | / | / | / |
| Valine | / | / | / | / | / | / | / | / | / | / | / | / | / | / | / | 1.36 | 0.80 | 0.02 |
| Vitamine K1 | / | / | / | / | / | / | 1.70 | 0.79 | 0.03 | 1.49 | -0.81 | 0.01 | / | / | / | / | / | / |
| α-Glucose | 1.55 | -0.77 | / | 1.43 | / | 0.03 | 1.43 | / | 0.04 | / | / | / | / | / | / | / | / | / |

^a^ Variable importance in projection. The VIP values at the top 20% were used as the cutoff values for the statistical significance.

^b^ Correlation coefficients, positive and negative signs indicate positive and negative correlation in the concentrations, respectively. The correlation coefficient of│r│> 0.666 was used as the cutoff value for the statistical significance based on the discrimination significance.

^c^ p-Value, obtained from t-test, p < 0.05 mean statistically significant difference.

^d^ ‘‘/’’ means the correlation coefficient│r│ is less than 0.666 or VIP value is not at the top 20%, or p>0.05.

**Table S3** Summary of metabolic variations in liver extracts induced by Fe@Si-NPs between different pairwise groups

| **Metabolites** | **C6-S6** | | | **C6-M6** | | | **C6-L6** | | | **C48-S48** | | | **C48-M48** | | | **C48-L48** | | |
| --- | --- | --- | --- | --- | --- | --- | --- | --- | --- | --- | --- | --- | --- | --- | --- | --- | --- | --- |
|  | **VIP**^a^ | **R**^b^ | **p**^c^ | **VIP** | **R** | **p** | **VIP** | **R** | **p** | **VIP** | **R** | **p** | **VIP** | **R** | **p** | **VIP** | **R** | **p** |
| 2-Hydroxyisobutyrate | /^d^ | / | / | 1.36 | / | 0.01 | / | / | / | 1.51 | / | 0.01 | / | / | / | / | / | / |
| 3-Methylhistidine | / | / | / | 1.47 | / | 0.02 | 1.56 | / | 0.00 | 1.41 | / | 0.02 | / | / | / | / | / | / |
| Acetate | 1.40 | / | 0.03 | 1.62 | / | 0.00 | 1.65 | / | 0.00 | 1.40 | / | 0.00 | / | / | / | / | / | / |
| Alanine | / | / | / | 1.62 | / | 0.00 | 1.32 | 0.76 | / | / | / | / | / | / | / | / | / | / |
| AMP | / | / | / | / | / | / | 1.44 | -0.81 | / | / | / | / | / | / | / | / | / | / |
| Aspartate | / | / | / | / | / | / | 1.49 | 0.89 | / | / | / | / | / | / | / | / | / | / |
| ATP | 1.65 | / | 0.00 | 1.51 | / | 0.01 | 1.46 | 0.88 | 0.02 | 1.42 | / | 0.04 | / | / | / | / | / | / |
| Betaine | 1.56 | / | 0.02 | / | / | / | / | / | / | / | / | / | / | / | / | / | / | / |
| Choline | / | / | / | 1.39 | 0.83 | 0.01 | / | / | / | / | / |  | / | / | / | / | / | / |
| Cytidine | / | / | / | / | / | / | / | / | / | 1.55 | / | 0.01 | / | / | / | / | / | / |
| Deoxyguanosine | 1.35 | / | 0.04 | 1.45 | 0.78 | 0.00 | 1.56 | / | 0.01 |  | / | / | / | / | / | / | / | / |
| Dimethylglycine | / | / | / | 1.37 | / | 0.04 | / | / | / | / | / | / | / | / | / | / | / | / |
| Deoxyuridine | / | / | / | / | / | / | / | / | / | / | / | / | 1.32 | 0.85 | / | 1.37 | 0.94 | / |
| Ethanolamine | 1.45 | 0.83 | / | / | / | / | / | / | / | / | / | / | / | / | / | / | / | / |
| Formate | 1.63 | / | 0.03 | 1.70 | 0.93 | 0.00 | 1.70 | 0.75 | 0.00 | 1.60 | / | 0.00 | / | / | / | / | / | / |
| Fumarate | / | / | / | / | 0.77 | 0.02 | / | / | / | 1.58 | 0.81 | 0.03 | 1.42 | 0.78 | / | 1.34 | / | / |
| Glycerol | 1.32 | / | 0.03 | 1.34 | / | 0.05 | / | / | / | / | 0.76 | 0.02 | / | / | / | / | / | / |
| Glycogen | 1.71 | / | 0.01 | 1.67 | 0.85 | 0.02 | 1.55 | 0.84 | / | 1.30 | / | 0.03 | / | / | / | / | / | / |
| Glutamine | / | / | / | 1.67 | 0.79 | / | 1.67 | / | 0.00 | / | / | / | / | / | / | / | / | / |
| Glutamate | 1.69 | / | 0.05 | 1.43 | / | 0.01 | 1.66 | / | 0.03 | / | / | / | / | / | / | / | / | / |
| Glutathione | 1.53 | 0.92 | 0.03 | 1.51 | 0.85 | 0.01 | 1.40 | 0.85 | / | / | / | / | / | / | / | 1.32 | -0.78 | / |
| Histidine | / | / | / | 1.35 | 0.89 | / | 1.58 | 0.97 | 0.01 | 1.44 | / | 0.01 | 1.46 | 0.85 | / | 1.37 | 0.90 | / |
| Isobutyrate | / | / | / | / | / | / | 1.53 | 0.84 | / | / | / | / | / | / | / | / | / | / |
| Lactate | / | / | / | 1.43 | / | 0.02 | / | / | / | / | / | / | 1.31 | -0.79 | / | 1.35 | -0.76 | / |
| Lysine | / | / | / | 1.58 | 0.83 | 0.05 | 1.58 | 0.86 | / | / | / | / | / | / | / | / | / | / |
| myo-Inositol | 1.53 | / | 0.05 | / | / | / | / | / | / | 1.38 | / | 0.04 | / | / | / | / | / | / |
| Nicotinamide | 1.76 | / | 0.03 | 1.68 | 0.84 | 0.00 | 1.65 | / | 0.00 | 1.70 | / | 0.00 | / | / | / | / | / | / |
| NAD | / | / | / | 1.68 | / | 0.00 | 1.67 | / | 0.00 | 1.74 | / | 0.00 | / | / | / | 1.58 | -0.82 | / |
| NADP | / | / | / | 1.67 | / | 0.00 | 1.70 | / | 0.00 | 1.70 | / | 0.00 | / | / | / | / | / | / |
| N-Acetylglutamate | 1.61 | / | 0.01 | 1.62 | / | 0.01 | / | / | / | / | / | / | / | / | / | / | / | / |
| Picolinate | 1.72 | / | 0.03 | 1.67 | / | 0.03 | / | / | / | / | / | / | / | / | / | 1.44 | 0.76 | / |
| Pantothenate | / | / | / | / | / | / | 1.61 | / | 0.02 | / | / | / | / | / | / | / | / | / |
| Phosphocholine | / | / | / | 1.54 | / | 0.00 | / | / | / | / | / | / | / | / | / | / | / | / |
| Pyroglutamate | / | / | / | 1.66 | / | 0.02 | 1.67 | / | 0.00 | / | / | / | 1.40 | -0.75 | / | / | / | / |
| Phenylalanine | / | / | / |  | / | / | / | / | / | 1.52 | / | 0.01 | / | / | / | / | / | / |
| Pterin | / | / | / | 1.55 | / | 0.01 | 1.40 | / | 0.01 | / | / | / | / | / | / | / | / | / |
| Pyruvate | / | / | / | 1.42 | / | 0.01 | / | / | / | / | / | / | / | / | / | / | / | / |
| Quinone | / | / | / | 1.38 | -0.75 | 0.01 | 1.43 | / | 0.01 | 1.47 | / | 0.02 | / | / | / | / | / | / |
| Sarcosine | 1.41 | -0.78 | / | / | -0.80 | 0.02 | / | / | / | / | / | / | / | / | / | / | / | / |
| Serine | 1.66 | 0.82 | 0.01 | 1.48 | / | 0.01 | / | / | / | / | / | / | 1.35 | 0.83 | / | / | / | / |
| Succinate | / | 0.78 | 0.05 | 1.60 | / | 0.00 | 1.44 | / | 0.00 | / | / | / | / | / | / | / | / | / |
| Threonine | 1.53 | / | 0.05 | 1.43 | 0.80 | 0.03 | 1.39 | 0.85 | / | / | / | / | / | / | / | / | / | / |
| Trimethylamine | / | / | / | 1.46 | 0.81 | / | / | / | / | / | / | / | / | / | / | / | / | / |
| Trigonelline | 1.37 | 0.76 | / | / | / | / | / | / | / | / | / | / | / | / | / | / | / | / |
| Tryptophan | 1.45 | 0.78 | / | 1.45 | / | 0.01 | 1.67 | / | 0.00 | / | / | / | / | / | / | / | / | / |
| Tyrosine | / | / | / | 1.34 | / | 0.04 | / | / | / | 1.53 |  | 0.01 | 1.50 | -0.81 | / | 1.43 | -0.91 | / |
| Unknown-3 | / | / | / | / | / | / | 1.31 | -0.82 | / | / | / | / | / | / | / | / | / | / |
| Unknown-4 | / | / | / | / | / | / | / | / | / | 1.42 | 0.85 |  | 1.39 | 0.92 | / | 1.36 | 0.81 | / |
| Unknown-9 | / | / | / | / | / | / | / | / | / | / | / | / | / | / | / | 1.39 | 0.91 | / |
| Urocanate | 1.53 | / | 0.05 | 1.47 | / | 0.00 | / | / | / | / | 0.78 | 0.04 | 1.39 | 0.90 | / | / | / | / |
| Uridine | 1.50 | -0.86 | / | 1.40 | -0.89 | / | / | / | / | 1.46 | / | 0.02 | / | / | / | / | / | / |
| Uridine diphosphate glucose | 1.56 | / | 0.03 | 1.53 | / | 0.01 | / | / | / | 1.30 | / | 0.05 | / | / | / | / | / | / |
| Valine | / | / | / | / | / | / | / | / | / | / | / | / | / | / | / | 1.40 | 0.80 | / |
| Vitamine K1 | / | / | / | / | / | / | 1.48 | / | 0.01 | / | / | / | / | / | / | / | / | / |
| Vitamine K3 | / | / | / | / | / | / | 1.41 | / | 0.04 | / | / | / | / | / | / | / | / | / |
| Xanthine | / | / | / | / | / | / | / | / | / | 1.50 | / | 0.01 | / | / | / | 1.49 | 0.79 | / |
| α-Glucose | 1.72 | / | 0.01 | 1.65 | / | 0.00 | 1.64 | / | 0.04 | / | / | / | 1.54 | 0.91 | / | 1.30 | 0.86 | / |
| β-Glucose | 1.74 | / | 0.00 | 1.63 | / | 0.00 | 1.58 | / | 0.02 | / | / | / | / | / | / | / | / | / |

^a^ Variable importance in projection. The VIP values at the top 20% were used as the cutoff values for the statistical significance.

^b^ Correlation coefficients, positive and negative signs indicate positive and negative correlation in the concentrations, respectively. The correlation coefficient of│r│> 0.666 was used as the cutoff value for the statistical significance based on the discrimination significance.

^c^ p-Value, obtained from t-test, p < 0.05 mean statistically significant difference.

^d^ ‘‘/’’ means the correlation coefficient│r│ is less than 0.666 or VIP value is not at the top 20%, or p>0.05.

**Table S4** Summary of metabolic variations in lung extracts induced by Fe@Si-NPs between different pairwise groups

| **Metabolites** | **C6-S6** | | | **C6-M6** | | | **C6-L6** | | | **C48-S48** | | | **C48-M48** | | | **C48-L48** | | |
| --- | --- | --- | --- | --- | --- | --- | --- | --- | --- | --- | --- | --- | --- | --- | --- | --- | --- | --- |
|  | **VIP**^a^ | **R**^b^ | **P**^c^ | **VIP** | **R** | **p** | **VIP** | **R** | **p** | **VIP** | **R** | **p** | **VIP** | **R** | **p** | **VIP** | **R** | **p** |
| 1-Methylhistidine | /^d^ | / | / | / | / | / | / | / | / | / | / | / | / | / | / | 1.48 | 0.80 | / |
| Acetate | 1.78 | 0.77 | / | / | / | / | 1.78 | 0.89 | / | / | / | / | / | / | / | 1.74 | 0.93 | / |
| Adenosine | / | / | / | / | / | / | / | / | / | / | / | / | / | / | / | 1.41 | -0.75 | / |
| Cholate | / | / | / | / | / | / | 1.53 | / | 0.01 | / | / | / | / | / | / | / | / | / |
| Creatine | 1.95 | 0.86 | / | / | / | / | 1.49 | / | 0.00 | / | / | / | / | / | / | 1.41 | / | 0.04 |
| Creatinine | / | / | / | 1.38 | / | 0.00 | 1.84 | 0.92 | 0.00 | 2.49 | 0.80 | / | / | / | / | 1.62 | 0.86 | / |
| Cytidine | 1.34 | / | 0.03 | / | / | / | / | / | / | / | / | / | / | / | / | / | / | / |
| Deoxyguanosine | / | / | / | 1.58 | / | 0.01 | 1.51 |  | 0.00 |  |  | / | / | / | / | 1.63 | / | 0.00 |
| Ethanol | / | / | / | / | / | / | / | / | / | / | / | / | 2.47 | -0.85 | / | / | / | / |
| Ethanolamine | 1.87 | 0.82 | / | / | / | / | / | / | / | / | / | / | / | / | / | 1.56 | -0.82 | / |
| Formate | / | / | / | 1.87 | 0.92 | / | 1.93 | 0.98 | / | 3.02 | 0.98 | / | 2.51 | 0.87 | 0.00 | 1.75 | 0.94 | 0.00 |
| Fumarate | / | / | / | 1.40 | / | 0.04 | / | / | / | / | / | / | 2.24 | / | 0.00 | / | / | / |
| Glutamine | / | / | / | / | / | / | / | / | / | / | / | / | / | / | / | 1.44 | -0.76 | / |
| Glutathione | 1.85 | -0.81 | / | / | / | / | / | / | / | 1.36 | / | 0.02 | / | / | / | 1.43 | -0.76 | / |
| Glycerophosphorylcholine | 1.75 | 0.76 | / | / | / | / | / | / | / | / | / | / | / | / | / | / | / | / |
| Glycogen | 2.00 | -0.86 | / | / | / | / | / | / | / | / | / | / | / | / | / | / | / | / |
| Inosine | 1.72 | 0.76 | / | / | / | / | / | / | / | / | / | / | / | / | / | / | / | / |
| Isoleucine | / | / | / | 1.32 | / | 0.04 | 1.45 | / | 0.00 | / | / | / | / | / | / | 1.32 | / | 0.00 |
| Lactate | 1.81 | 0.80 | / | / | / | / | / | / | / | / | / | / | / | / | / | / | / | / |
| Leucine | / | / | / | / | / | / | / | / | / | / | / | / | / | / | / | 1.75 | 0.94 | / |
| Lysine | / | / | / | / | / | / | 1.58 | -0.80 | / | / | / | / | / | / | / | 1.49 | 0.79 | / |
| myo-Inositol | / | / | / | 1.57 | 0.77 | / | 1.87 | 0.95 | / | / | / | / | / | / | / | 1.54 | 0.82 | / |
| N-Acetylglutamate | / | / | / | / | / | / | / | / | / | / | / | / | / | / | / | 1.56 | 0.84 | 0.00 |
| Nicotinamide | / | / | / | / | / | / | / | / | / | / | / | / | / | / | / | 1.31 | / | 0.00 |
| NAD | / | / | / | / | / | / | / | / | / | / | / | / | / | / | / | 1.56 | / | 0.00 |
| Pantothenate | 1.93 | 0.85 | / | 1.89 | 0.93 | / | 1.86 | 0.93 | / | / | / | / | / | / | / | 1.65 | 0.87 | 0.00 |
| Phenylalanine | / | / | / | 1.54 | 0.79 | / | / | / | / | / | / | / | / | / | / | 1.53 | 0.79 | 0.00 |
| Propionate | / | / | / | 1.65 | 0.79 | / | / | / | / | / | / | / | 2.47 | 0.85 | / | 1.53 | 0.81 | 0.01 |
| Pyroglutamate | / | / | / | / | / | / | / | / | / | / | / | / | / | / | / | 1.59 | 0.83 | 0.00 |
| Sarcosine | / | / | / | / | / | / | 1.46 | / | 0.04 | / | / | / | / | / | / | / | / | / |
| Succinate | / | / | / | / | / | / | 1.44 | / | 0.02 | 2.70 | 0.87 | / | / | / | / | 1.57 | 0.84 | 0.01 |
| Tryptophan | 1.97 | -0.87 | / | / | / | / | 1.42 | / | 0.04 | / | / | / | / | / | / | 1.30 | / | 0.01 |
| Unknown-13 | / | / | / | / | / | / | 1.51 | 0.76 | / | / | / | / | / | / | / | / | / | / |
| Unknown-5 | / | / | / | / | / | / | 1.63 | 0.81 | / | / | / | / | 2.45 | 0.84 | / | 1.80 | 0.96 | / |
| Uridine | / | / | / | / | / | / | / | / | / | / | / | / | / | / | / | 1.35 | / | 0.00 |
| Vitamine K1 | / | / | / | 1.82 | 0.89 | / | 1.84 | 0.92 | 0.01 | / | / | / | / | / | / | 1.78 | 0.96 | 0.01 |
| Xanthine | / | / | / | / | / | / | / | / | / | / | / | / | / | / | / | 1.33 | / | 0.02 |

^a^ Variable importance in projection. The VIP values at the top 20% were used as the cutoff values for the statistical significance.

^b^ Correlation coefficients, positive and negative signs indicate positive and negative correlation in the concentrations, respectively. The correlation coefficient of│r│> 0.666 was used as the cutoff value for the statistical significance based on the discrimination significance.

^c^ p-Value, obtained from t-test, p < 0.05 mean statistically significant difference.

^d^ ‘‘/’’ means the correlation coefficient│r│ is less than 0.666 or VIP value is not at the top 20%, or p>0.05.

**Table S5** Summary of metabolic variations in spleen extracts induced by Fe@Si-NPs between different pairwise groups

| **Metabolites** | **C6-S6** | | | **C6-M6** | | | **C6-L6** | | | **C48-S48** | | | **C48-M48** | | | **C48-L48** | | |
| --- | --- | --- | --- | --- | --- | --- | --- | --- | --- | --- | --- | --- | --- | --- | --- | --- | --- | --- |
|  | **VIP**^a^ | **R**^b^ | **P**^c^ | **VIP** | **R** | **p** | **VIP** | **R** | **p** | **VIP** | **R** | **p** | **VIP** | **R** | **p** | **VIP** | **R** | **p** |
| 1-Methylhistidine | /^d^ | / | / | 1.52 | -0.77 | / | 1.51 | -0.77 | / | 2.06 | -0.79 | / | / | / | / | / | / | / |
| 3-Hydroxybutyrate | / | / | / | 1.18 | / | 0.00 | / | / | / | / | / | / | 1.76 | -0.84 | / | 1.62 | -0.80 | / |
| Acetate | / | / | / | / | / | / | 1.69 | 0.92 | 0.00 | / | / | / | 1.47 | / | 0.04 | 1.64 | 0.95 | 0.00 |
| Adenosine monophosphate | / | / | / | 1.50 | / | 0.03 | / | / | / | / | / | / | / | / | / | 1.61 | -0.93 | / |
| Alanine | / | / | / | / | / | / | / | / | / | / | / | / | 1.52 | 0.77 | / | / | / | / |
| Aspartate | / | / | / | 1.63 | 0.87 | / | 1.59 | 0.88 | / | / | / | / | / | / | / | 1.63 | 0.93 | / |
| Choline | 1.49 | 0.77 | / | 1.61 | 0.86 | / | 1.67 | 0.80 | / | / | / | / | / | / | / | / | / | / |
| Creatine | / | / | / | 1.58 | -0.83 | / | 1.63 | -0.89 | / | / | / | / | 1.61 | -0.82 | / | / | / | / |
| Cytidine | / | / | / | 1.09 | / | 0.05 | / | / | / | / | / | / | / | / | / | / | / | / |
| Dihydrothymine | / | / | / | / | / | / | 1.46 | 0.78 | / | / | / | / | 1.53 | 0.78 | / | 1.62 | 0.94 | / |
| Ethanol | / | / | / | / | / | / | / | / | / | / | / | / | 1.63 | -0.83 | 0.03 | 1.38 | -0.79 | 0.04 |
| Ethanolamine | / | / | / | 1.77 | -0.94 | / | 1.69 | / | 0.01 | / | / | / | / | / | / | / | / | / |
| Formate | 1.63 | 0.88 | 0.00 | 1.65 | 0.88 | 0.00 | 1.77 | 0.98 | 0.00 | 2.37 | 0.92 | 0.00 | 1.78 | 0.92 | 0.00 | 1.65 | 0.95 | 0.00 |
| Fumarate | / | / | / | 1.28 | / | / | / | / | / | / | / | / | / | / | / | / | / | / |
| Glutamate | 1.53 | 0.78 | / | 1.57 | 0.83 | / | / | / | / | / | / | / | 1.61 | -0.77 | / | 1.58 | / | / |
| Glutamine | 1.60 | -0.86 | / | 1.75 | -0.93 | / | 1.79 | -0.98 | 0.02 | / | / | / | 1.78 | -0.91 | / | 1.61 | -0.92 | 0.02 |
| Glutathione | / | / | / | 1.62 | 0.85 | / | 1.55 | 0.85 | / | / | / | / | / | / | / | 1.61 | 0.93 | 0.03 |
| Glycerol | / | / | / | / | / | / | / | / | / | / | / | / | / | / | / | 1.42 | 0.75 | 0.01 |
| Glycerophosphorylcholine | / | / | / | / | / | / | / | / | / | / | / | / | / | / | / | / | -0.86 | 0.00 |
| Glycogen | 1.62 | -0.84 | 0.04 | / | / | / | / | / | / | 1.97 | -0.75 | / | / | / | / | / | / | / |
| Inosine | / | / | / | / | / | / | / | / | / | / | / | / | / | / | / | / | / | / |
| Isoleucine | 1.48 | -0.76 | 0.04 | 1.60 | -0.83 | 0.04 | 1.59 | -0.84 | / | / | / | / | 1.54 | 0.76 | / | 1.58 | 0.91 | 0.01 |
| Lactate | / | / | / | 1.49 | -0.79 | / | 1.46 | -0.80 | / | / | / | / | / | / | / | / | / | / |
| Leucine | 1.46 |  | 0.03 | 1.50 | -0.78 | / | / | / | / | / | / | / | / | / | / | 1.41 | 0.81 | 0.00 |
| Lysine | / | / | / | / | / | / | 1.72 | 0.95 | / | 2.08 | 0.80 | 0.00 | 1.78 | 0.92 | / | 1.65 | 0.94 | / |
| Malonate | / | / | / | / | / | / | 1.38 | -0.76 | 0.03 | / | / | / | / | / | / | / | / | / |
| myo-Inositol | / | / | / | / | / | / | 1.64 | 0.83 | / | / | / | / | / | / | / | 1.56 | 0.76 | / |
| N-Acetylglutamate | / | / | / | / | / | / | 1.46 | 0.77 | / | / | / | / | 1.74 | 0.90 | / | 1.63 | 0.94 | / |
| Nicotinamide | 1.66 | 0.89 | / | 1.82 | 0.97 | / | 1.49 | 0.79 | / | 1.92 | 0.75 | 0.00 | 1.58 | 0.80 | 0.00 | 1.52 | -0.87 | 0.00 |
| NAD | / | / | / | 1.63 | -0.86 | 0.03 | 1.75 | -0.77 | 0.01 | 1.83 | / | 0.00 | 1.56 | / | 0.00 | 1.54 | / | 0.00 |
| NADP | 1.62 | 0.86 | / | / | / | / | / | / | / | / | / | / | / | / | / | / | / | / |
| Pantothenate | / | / | / | / | / | / | 1.60 | 0.87 | 0.02 | / | / | / | 1.54 | 0.77 | / | 1.54 | 0.88 | 0.01 |
| Phenylalanine | / | / | / | 1.67 | 0.90 | / | 1.76 | 0.97 | / | / | / | / | 1.70 | 0.88 | 0.01 | 1.60 | 0.92 | 0.00 |
| Propionate | 1.67 | -0.89 | / | / | / | / | 1.49 | 0.82 | / | 1.99 | 0.76 | / | 1.69 | 0.87 | / | 1.63 | 0.94 | 0.00 |
| Pyroglutamate | / | / | / | 1.75 | 0.93 | / | 1.79 | 0.99 | / | 1.98 | 0.77 | / | 1.71 | 0.87 | 0.00 | 1.60 | 0.93 | / |
| Quinone | / | / | / | / | / | / | / | / | / | / | / | / | / | / | / | 1.37 | 0.79 | 0.04 |
| Sarcosine | / | / | / | 1.07 | / | 0.04 | / | / | / | / | / | / | / | / | / | 1.52 | 0.85 | / |
| Serine | / | / | / | 1.68 | -0.89 | / | / | / | / | / | / | / | 1.68 | -0.87 | / | 1.60 | -0.93 | / |
| Succinate | / | / | / | / | / | / | 1.54 | 0.84 | 0.02 | / | / | / | / | / | / | / | / | / |
| Trimethylamine | / | / | / | / | / | / | / | / | / | / | / | / | / | / | / | 1.31 | 0.75 | 0.00 |
| Tryptophan | 1.55 | -0.77 | / | 1.63 | 0.84 | / | 1.66 | 0.91 | / | / | / | / | 1.79 | 0.91 | 0.00 | 1.64 | 0.95 | 0.00 |
| Tyrosine | / | / | / | 1.50 | -0.76 | / | / | / | / | / | / | / | 1.32 | / | 0.00 | 1.64 | 0.95 | 0.00 |
| Unknown-1 | / | / | / | / | / | / | / | / | / | / | / | / | / | / | / | 1.57 | -0.91 | / |
| Unknown-11 | / | / | / | / | / | / | / | / | / | / | / | / | 1.54 | 0.77 | / | / | / | / |
| Unknown-12 | 1.45 | 0.76 | / | / | / | / | / | / | / | / | / | / | / | / | / | / | / | / |
| Unknown-5 | / | / | / | / | / | / | 1.67 | 0.91 | / | / | / | / | 1.71 | 0.88 | / | 1.63 | 0.94 | / |
| Unknown-6 | / | / | / | / | / | / | 1.59 | -0.87 | / | / | / | / | / | / | / | / | / | / |
| Uridine | / | / | / | / | / | / | / | / | / | / | / | / | 1.44 | / | 0.04 | / | / | / |
| Uridine diphosphate glucose | 1.62 | -0.87 | / | / | / | / | / | / | / | / | / | / | 1.40 | / | / | 1.37 | -0.79 | / |
| Valine | 1.53 | -0.79 | / | / | / | / | 1.47 | -0.76 | / | / | / | / | / | / | / | / | / | / |
| α-Glucose | / | / | / | 1.15 | / | 0.03 | 1.59 | -0.88 | 0.04 | / | / | / | / | / | / | / | / | / |

^a^ Variable importance in projection. The VIP values at the top 20% were used as the cutoff values for the statistical significance.

^b^ Correlation coefficients, positive and negative signs indicate positive and negative correlation in the concentrations, respectively. The correlation coefficient of│r│> 0.666 was used as the cutoff value for the statistical significance based on the discrimination significance.

^c^ p-Value, obtained from t-test, p < 0.05 mean statistically significant difference.

^d^ ‘‘/’’ means the correlation coefficient│r│ is less than 0.666 or VIP value is not at the top 20%, or p>0.05.
